# Transcriptome analysis of host-associated differentiation in *Bemisia tabaci* (Hemiptera: Aleyrodidae)

**DOI:** 10.3389/fphys.2014.00487

**Published:** 2014-12-10

**Authors:** Wen Xie, Qingjun Wu, Shaoli Wang, Xiaoguo Jiao, Litao Guo, Xuguo Zhou, Youjun Zhang

**Affiliations:** ^1^Department of Plant Protection, Institute of Vegetables and Flowers, Chinese Academy of Agricultural SciencesBeijing, China; ^2^Department of Entomology, S-225 Agricultural Science Center North, University of KentuckyLexington, KY, USA

**Keywords:** *Bemisia tabaci*, comparative transcriptomic analysis, host-associated differentiation, qRT-PCR, *Cathepsin*

## Abstract

Host-associated differentiation is one of the driving forces behind the diversification of phytophagous insects. In this study, host induced transcriptomic differences were investigated in the sweetpotato whitefly *Bemisia tabaci*, an invasive agricultural pest worldwide. Comparative transcriptomic analyses using coding sequence (CDS), 5′ and 3′ untranslated regions (UTR) showed that sequence divergences between the original host plant, cabbage, and the derived hosts, including cotton, cucumber and tomato, were 0.11–0.14%, 0.19–0.26%, and 0.15–0.21%, respectively. In comparison to the derived hosts, 418 female and 303 male transcripts, respectively, were up-regulated in the original cabbage strain. Among them, 17 transcripts were consistently up-regulated in both female and male whiteflies originated from the cabbage host. Specifically, two ESTs annotated as *Cathepsin B* or *Cathepsin B-like* genes were significantly up-regulated in the original cabbage strain, representing a transcriptomic response to the dietary challenges imposed by the host shifting. Results from our transcriptome analysis, in conjunction with previous reports documenting the minor changes in their reproductive capacity, insecticide susceptibility, symbiotic composition and feeding behavior, suggest that the impact of host-associated differentiation in whiteflies is limited. Furthermore, it is unlikely the major factor contributing to their rapid range expansion/invasiveness.

## Introduction

Host-associated differentiation (HAD) in insects, occurring mainly in sympatric populations, is generally considered to be an adaptation to different habitats and resources. This adaptive response plays a crucial role in the early stage of speciation and may lead to reproductive isolation (Nosil et al., 2002; Rundle and Nosil, 2005; Stireman et al., 2005; Schluter, 2009). The advent of Genomics Era, especially the development of the next generation sequencing technology (NGS) (Mardis, 2008; Schuster, 2008), provides an unprecedented opportunity for us to understand HAD at the omics level and its potential association with the host-derived genetic divergence.

The sweetpotato whitefly *Bemisia tabaci* (Gennadius) (Hemiptera: Aleyrodidae), an invasive agricultural pest, causes severe damages to agricultural and horticultural crops worldwide through direct feeding and indirect vectoring of plant viruses (Jones, 2003). More than 34 genetically distinct, yet morphologically indistinguishable *B. tabaci* species have been identified (Perring, 2001; Dinsdale et al., 2010; Elbaz et al., 2010; De Barro et al., 2011; De Barro, 2012; Liu et al., 2012). Among them, *B. tabaci* B (also known as the Middle East-Asia Minor 1) has emerged as a major insect pest due to their global invasion for the past 20 years (Brown et al., 1995; Liu et al., 2007). In China, native *B. tabaci* was first recorded in the late 1940s. However, *B. tabaci* has not being a pest for vegetable, fruit and ornamental crops until the introduction and establishment of the invasive *B. tabaci* B in the mid-1990s (Zhang et al., 2005; Chu et al., 2006).

In recent years, several studies have been focused on the comparative analysis of host adaptation among *B. tabaci* species complex (Iida et al., 2009; Tsueda and Tsuchida, 2011; Jiao et al., 2012, 2013; Saleh et al., 2012). Species-specific divergence can be attributed to the host plant's response to feeding, and further mediated by the interactions among *B. tabaci*, endosymbionts, plant viruses and natural enemies (Lapidot et al., 2001; Colvin et al., 2006; Inbar and Gerling, 2008; Pan et al., 2012). The performance of *B. tabaci* B on various host plants, including cotton, tobacco, cabbage, squash, kidney beans, garden beans and soybeans, were evidently different (Zang et al., 2006; Mansaray and Sundufu, 2009). The greenhouse whitefly showed substantially different feeding behavior in association with host-plant acceptance (Lei et al., 1998). Whitefly cryptic species maintained on different host plants exhibited differential susceptibilities to insecticides (Liang et al., 2007; Castle et al., 2009; Xie et al., 2011). Host plants themselves may play an important role in explaining why morphologically- indistinguishable *B. tabaci* sibling species have different host range (Brown et al., 1995).

Originated from a *B. tabaci* B population collected in 2004, we established several laboratory whitefly strains feeding exclusively on their original host, cabbage, and the derived hosts, cucumber, tomato, cotton and poinsettia. After maintaining on their respective hosts for 3–5 years, these whitefly strains displayed few variations in their life history traits (Lou et al., 2005; Fu et al., 2008), insecticide susceptibility (Xie et al., 2011), symbiotic contents (Pan et al., 2013) and feeding behavior (Liu et al., 2013). Specifically, *B. tabaci* strains maintained on cabbage and poinsettia had a significantly longer developmental time from egg to adult than the tomato strain (Lou et al., 2005; Fu et al., 2008). In terms of insecticide susceptibility, a whitefly strain derived from poinsettia was, in general, less sensitive to insecticide treatments (Xie et al., 2011). As for the symbiont composition, the cucumber strain harbored more *Portiera* than strains maintained on cabbage and cotton, while cabbage strain harbored more *Rickettsia* and *Cardinium* (Pan et al., 2013). Using electrical penetration graph technology to quantitatively measure the feeding behavior, we showed that *B. tabaci* maintained on their original cabbage host outperformed whiteflies derived from cucumber and tomato (Liu et al., 2013). Despite these phenotypic differences between the original and derived hosts, genetic variations among whiteflies has yet been investigated, especially at the transcriptome level. In this study, we examined the sequence divergence between a *B. tabaci* population originated from a cabbage host and three other whitefly populations which have been subjected to the long-term isolation in cotton, cucumber and tomato, respectively, for multiple generations. Results from the comparative transcriptomic profiling will shed light on the molecular mechanisms underlying the HAD, and contribute to our understanding of the host-induced evolution in this emerging global invasive pest.

## Materials and methods

### Sample preparation

The parental whitefly population was established in 2004 by releasing 10 pairs of *B. tabaci* adults, which had been collected from cabbage in Haidian District of Beijing, into one screen cage containing one cabbage plant in a glasshouse (Xie et al., 2011, 2014; Liu et al., 2013; Pan et al., 2013). This original population was identified as a B-type (Middle East-Asia Minor 1) based on the mitochondria cytochrome oxidase 1 (mtCO1) marker (Zhang et al., 2005). After two generations, more cabbage plants (i.e., three plants in total) were placed into the parental population cage. At the fourth generation, each cabbage plant was randomly transferred from the parental population to a cage with one derived hosts, cotton, cucumber and tomato, respectively. These four *B. tabaci* populations, including one original and three derived, have the same genetic background and have been maintained in the same glasshouse without exposure to any insecticides for at least 4 years (approximately 60 generations) (Xie et al., 2011, 2014; Liu et al., 2013; Pan et al., 2013). Only 2-day-old adults of these different host strain female and male samples were collected respectively (about 1000 female or male from each sample), pooled into a plastic tube using an aspirator, and then snap frozen in liquid nitrogen, and transferred to a −80°C for freezer for the long-term storage.

### RNA isolation, cDNA library construction, and illumina sequencing

RNA from each female or male sample group was extracted, RNA purity and degradation were checked on 1% agarose gels, and further integrity was also confirmed using the 2100 Bioanalyzer (Agilent Technologies) with a minimum RNA integrated number value of 8. Poly (A)-containing RNA was then obtained from total RNA with the Dynabeads® mRNA purification kit (Invitrogen). Then, the mRNA was fragmented into small pieces using divalent cations under elevated temperature and the cleaved RNA fragments were used for first strand cDNA synthesis using reverse transcriptase and random primers. This was followed by second strand cDNA synthesis using DNA polymerase I and RNaseH. These cDNA fragments were subjected to end repair process and ligation of adapters. These products were purified and enriched with PCR to create the final cDNA library. All eight paired-end cDNA libraries (500 bp size), including female *B. tabaci* from cabbage (Caf), cucumber (Cuf), tomato (Tof), cotton (Cof), and male whiteflies from cabbage (Cam), cucumber (Cum), tomato (Tom), and cotton (Com), were prepared following the manufacturer's recommendations and sequenced on an Illumina GAII platform.

### Sequence divergence analysis

Sequence divergence analysis pipeline was modified from Wang et al. (2011) and Xie et al. (2014). Raw reads of eight cDNA libraries were all filtered to remove adaptor sequences and low quality sequences (containing reads with unknown sequences “N” and lower than 10 of mean phred scores). Then, the reads obtained from each library were assembled separately, and all pooled reads of 8 libraries were also assembled into a reference transcriptome using Trinity with default parameters. Pooled reference transcriptome was annotated using a BLAST search against the non-redundant (NR) database in NCBI, SWISS-PROT, KEGG and COG with an *e*-value cut-off of 1e^−5^.

A customized informatics pipeline was developed to calculate the divergence ratio between the original cabbage population and three derived host populations (cotton, cucumber and tomato). A bidirectional best hit method was used in BLAST search (blastn, *e*-value cutoff is 1e^−10^) to identify genes that are putatively pairs of reciprocal best matched sequences (RBMs) in the eight separately assembled cDNA libraries. Only pairs of sequences that corresponded unambiguously to the same ESTs in the pooled reference transcriptome, and were annotated to the same protein in Swissprot database with an *E* < 1 × 10^−5^, were selected as orthologs. Coding sequences (CDS) of the orthologous genes corresponding to the ESTs from the pooled reference transcriptome were determined by BLASTx against all known proteins in Swissprot database using a threshold of 1 × 10^−5^. CDS with unexpected stop codon in the Blast hit region and/or shorter than 150 bp was removed. Boundaries defining the primary structure of sequences, including the start and codon, 3′ and 5′UTR of each pair of orthologs, were predicted following Wang et al. (2011). Based on the extracted CDS, 5′UTR and 3′UTR of each pair of orthologs from each comparing group, sequence divergence was calculated by dividing the number of substitutions by the number of base pairs compared, while substitution rates were estimated separately for synonymous (Ks) and non-synonymous sites (Ka) using an approximate method implemented in the KaKs Calculator Version 1.2 with pair-wise approximate analyses (Yang and Nielsen, 2000; Tiffin and Hahn, 2002; Zhang et al., 2006; Wang et al., 2011).

### Identification of differentially expressed genes

Differentially expressed genes were determined according to Xie et al. (2014). Briefly, based on the number of assembled genes, trinity pooled transcripts and raw reads for each sample were analyzed using RSEM (v1.1.15) (Li and Dewey, 2011). To calculate the normalized read counts for each library, RNA composition bias and normalization factors were taken into account and calculated by edgeR (Robinson et al., 2010). The spearman correlation coefficient for all samples was generated with *heatmap 2* in R package (Durinck et al., 2009). The normalized read counts were subjected to the DEGseq package to identify differentially expressed genes using MARS model (*Q* < 0.05 and Fold Change >2) (Storey and Tibshirani, 2003; Wang et al., 2010).

### Quantitative real time PCR (qRT-PCR) analysis

A total of 300 newly emerged *B. tabaci* adults from the original cabbage (Ca) and derived cucumber (Cu), cotton (Co) and tomato (To) hosts were, respectively, collected and snap frozen in liquid nitrogen for the subsequent qRT-PCR analysis (representing three biological replicates, each using 100 adults). Total RNA was extracted using Trizol (Invitrogen) following the manufacturer's protocol. The resultant total RNA was re-suspended in nuclease-free water and the concentration was measured using Nanodrop (Thermo Scientific Nanodrop 2000). Approximately 0.5 μg of total RNA was used to synthesize the first-strand cDNA using a PrimerScript RT reagent Kit (TaKaRa). The resultant cDNA was diluted to 0.1 μg/μl for qRT-PCR analysis (ABI 7500) using a SYBR Green Realtime PCR Master Mix (TaKaRa). qRT-PCR primers were designed using the Primer Express 2.0 software. The cycling parameter was 95°C for 30 s, followed by 40 cycles of 95°C for 5 s and 62°C for 34 s, and concluded with the melting curve analysis (65–95°C in increments of 0.5°C every 5 s) to check for non-specific product amplification. Relative gene expression was calculated by the ΔCT method using *RPL29* as a reference (Li et al., 2013) to eliminate sample-to-sample variations in the initial cDNA samples.

## Result and discussion

### Genetic divergence among different host strains

**Figure 1:**
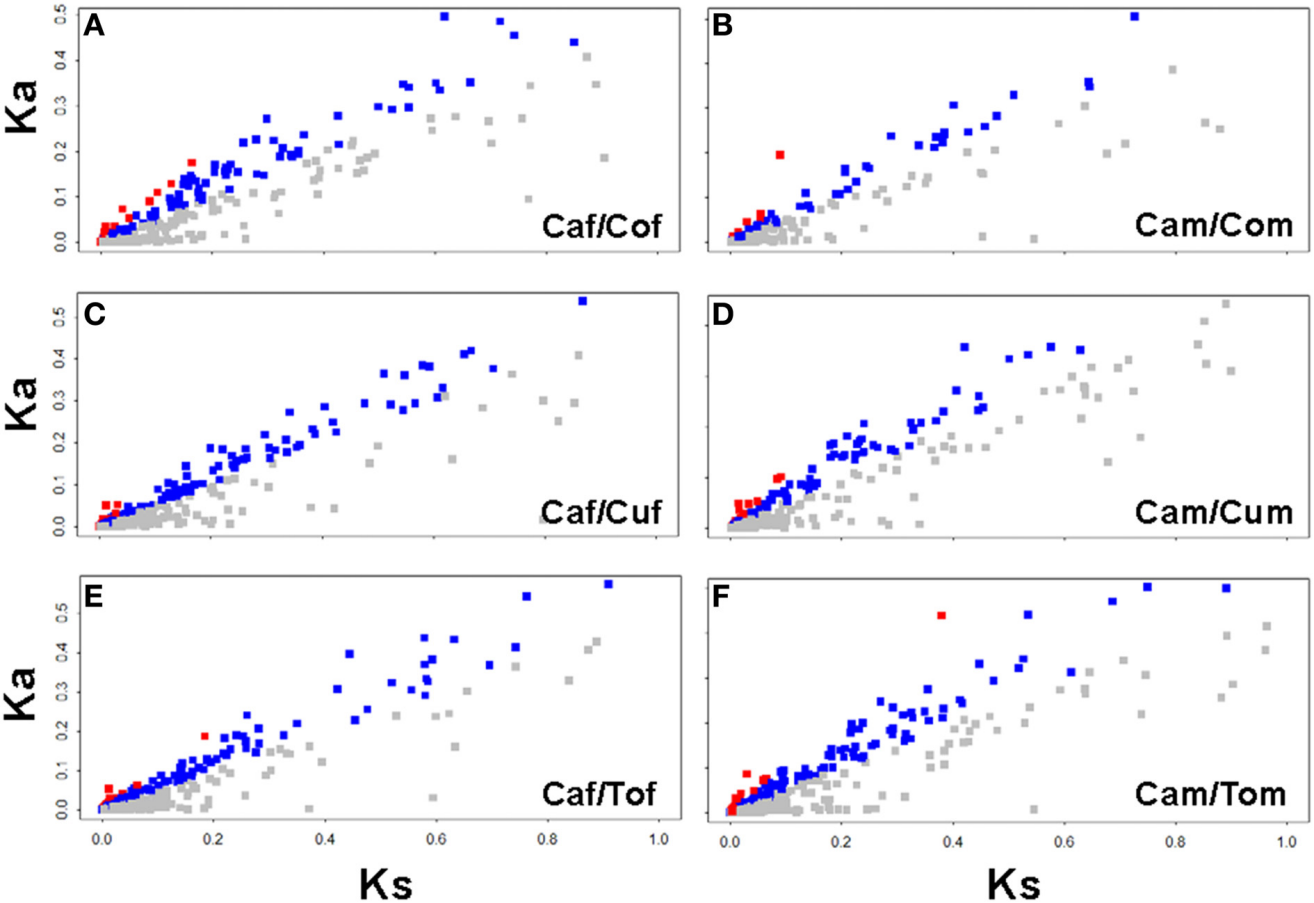
**Distribution of Ka and Ks in each host pairs**. Sequences with Ka/Ks ratio >1, in between 0.5 and 1, and <0.5 were highlighted in red, blue, and gray, respectively **(A)** Cabbage female vs. cotton female; **(B)** Cabbage female vs. cucumber female; **(C)** Cabbage female vs. tomato female; **(D)** Cabbage male vs. cotton male; **(E)** Cabbage male vs. cucumber male; and **(F)** Cabbage male vs. tomato male. Data analysis was carried out following Yang and Nielsen (2000) and Wang et al. (2012).

To compare the sequence divergence between *B. tabaci* maintained on their original host, cabbage, and their derived hosts, cotton, cucumber and tomato, respectively, we analyzed the orthologous gene pairs between their transcriptomes using a bidirectional best hit approach. As a result, 26020, 32193, 31222, 17916, 18678, and 39541 pairs of reciprocal best matched sequences corresponding, respectively, to Caf/Cof, Caf/Cuf, Caf/Tof, Cam/Com, Cam/Cum, and Cam/Tom pairing groups were identified (Table 1). Among them, 6718 (Caf/Cof), 7914 (Caf/Cuf), 7756 (Caf/Tof), 3642 (Cam/Com), 4920 (Cam/Cum), and 8031 (Cam/Tom) orthologous genes were annotated to the same protein in Swissprot database and corresponded to the same EST in the pooled reference transcriptome. In addition, 651 pairs of 5′UTR and 757 pairs of 3′UTR were identified in Caf/Cof, followed by 965 pairs of 5′UTR and 1323 pairs of 3′UTR in Caf/Cuf, 900 pairs of 5′UTR and 1162 pairs of 3′UTR in Caf/Tof, 200 pairs of 5′UTR and 266 pairs of 3′UTR in Cam/Com, 305 pairs of 5′UTR and 372 pairs of 3′UTR in Cam/Cum, 705 pairs of 5′UTR and 971 pairs of 3′UTR in Cam/Tom. The mean value of 5′UTR, CDS, 3′UTR of orthologous genes was 0.23% (0.19–0.26%), 0.13% (0.11–0.14%) and 0.19% (0.15–0.21%), respectively (Table 1). To identify genes undergoing positive and purifying selections due to the host induction, we estimated rates of non-synonymous (Ka) and synonymous (Ks) substitutions of cotton, cucumber and tomato with their original cabbage strain. If Ka/Ks >1, it is considered to be a positive selection, whereas when Ka/Ks <1, it is a purifying selection. We identified 24 (Caf/Cof), 17 (Caf/Cuf), 23 (Caf/Tof), 9 (Cam/Com), 13 (Cam/Cum), and 36 genes (Cam/Tom) with Ka/Ks ratio greater than one (Table 1; Tables S3–S8; Figure 1).

**Table 1:** **Divergence analysis between the original host and the derived hosts**.

Host Pairs^*^	**Matched sequences**	**Orthologs**	5′UTR (%)^**^	**CDS (%)**	**3′UTR (%)**	Ka/Ks >1^***^
Caf/Cof	26020	6718	651 (0.19)	6673 (0.13)	757 (0.20)	24
Caf/Cuf	32193	7914	965 (0.22)	7873 (0.11)	1323 (0.21)	17
Caf/Tof	31222	7756	900 (0.24)	7697 (0.11)	1162 (0.20)	23
Cam/Com	17916	3642	201 (0.26)	3608 (0.14)	266 (0.19)	9
Cam/Cum	18678	4920	305 (0.23)	4878 (0.14)	372 (0.15)	13
Cam/Tom	39541	8031	705 (0.22)	7958 (0.12)	971 (0.17)	36

* *Ca, Co, Cu, and To denote the original host plant cabbage, and the derived host plants cotton, cucumber, and tomato, respectively. Comparative analysis was carried out in both male (m) and female (f) whiteflies*.

** *The number of transcripts has 5′UTR (the average divergence rate of all 5′UTR)*.

*** *The number of orthologs if Ka (non-synonymous substitution rate)/Ks (synonymous substitution rate) >1*.

*Bemisia tabaci* B has more than 600 known host plants (Oliveira et al., 2001); however, few studies have explored the host inducted genetic variation using a next generation sequencing approach. Previous research associated with the genetic divergence in *B. tabaci* were mainly focused on the comparisons of *B. tabaci* samples from different geographical regions using a suite molecular markers, such as *mitochondrial 16S DNA, cytochrome oxidase 1* and the *nuclear ribosomal intergenic spacer* (Dinsdale et al., 2010; De Barro et al., 2011). Moreover, the differences among three cryptic species *B. tabaci* B, Q and Asia II 3 were revealed by the whole transcriptome divergence analysis (Wang et al., 2011, 2012). In this study, we indentified thousands of ortholog gene pairs and determined the whole sequence divergence between the original cabbage strain and the derived host strains (cotton, cucumber and tomato; >4 years) to be 0.11–0.14%, 0.19–0.26%, and 0.15–0.21%, respectively, for the CDS, 5′UTR and 3′UTR (Table 1). This value is lower than the reported mean divergence between human and chimpanzee (0.45, 1.12, and 0.86%, respectively, for CDS, 5′UTR and 3′UTR divergence) (Hellmann et al., 2003; Shi et al., 2003). It also lower than the ratio between *B. tabaci* B and Q (0.83, 1.66, and 1.43% respectively for CDS, 5′UTR and 3′UTR divergence) (Wang et al., 2011), between *B. tabaci* B and Asia II 3 (1.73% for CDS) and between *B. tabaci* Q and Asia II 3 (1.84% for CDS) (Wang et al., 2012). Above results indicate that the divergence between the original cabbage strain and derived host strains (cotton, cucumber and tomato) was limited between their transcriptomes, although these whiteflies have been subjected to the long-term isolation in different host plants (>4 years).

### Gene expression profile among different host strains

**Figure 2:**
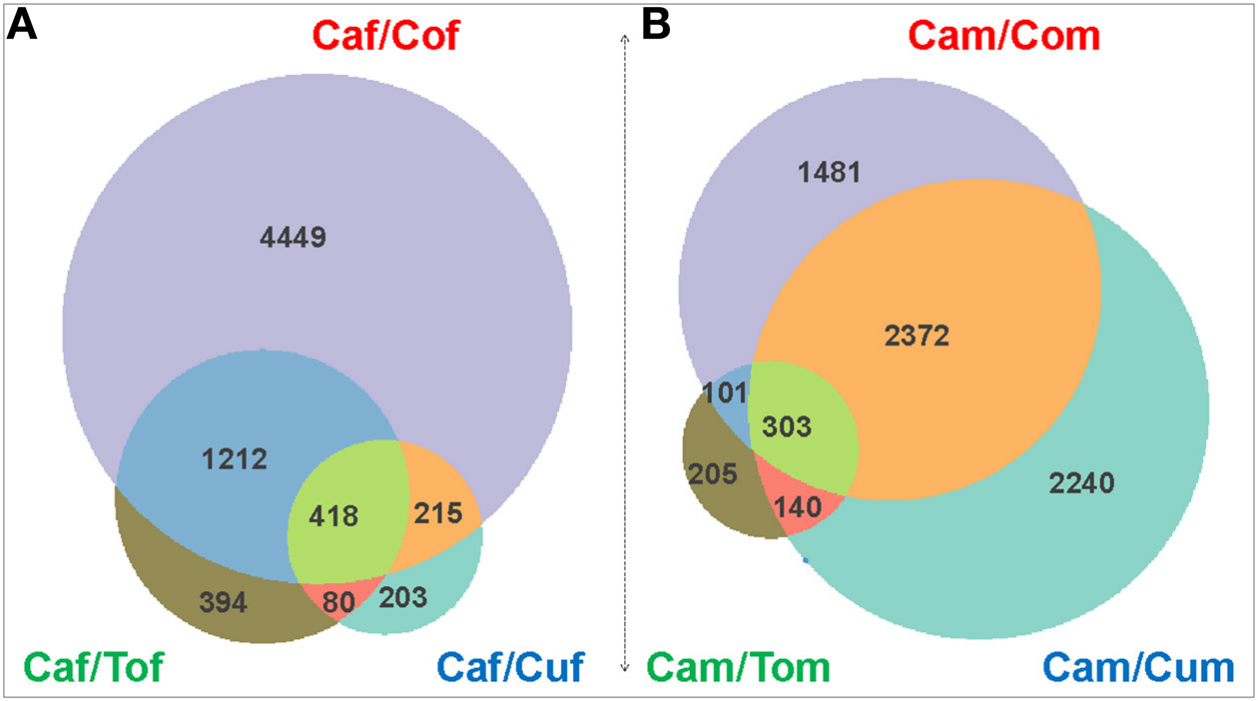
**Up-regulated genes between the original and the derived hosts**. Venn diagrams showed up-regulated genes in adult females **(A)** and males **(B)**. Ca, Co, Cu, and To denote the original host plant, cabbage, and derived host plants cotton, cucumber, and tomato, respectively.

**Figure 3:**
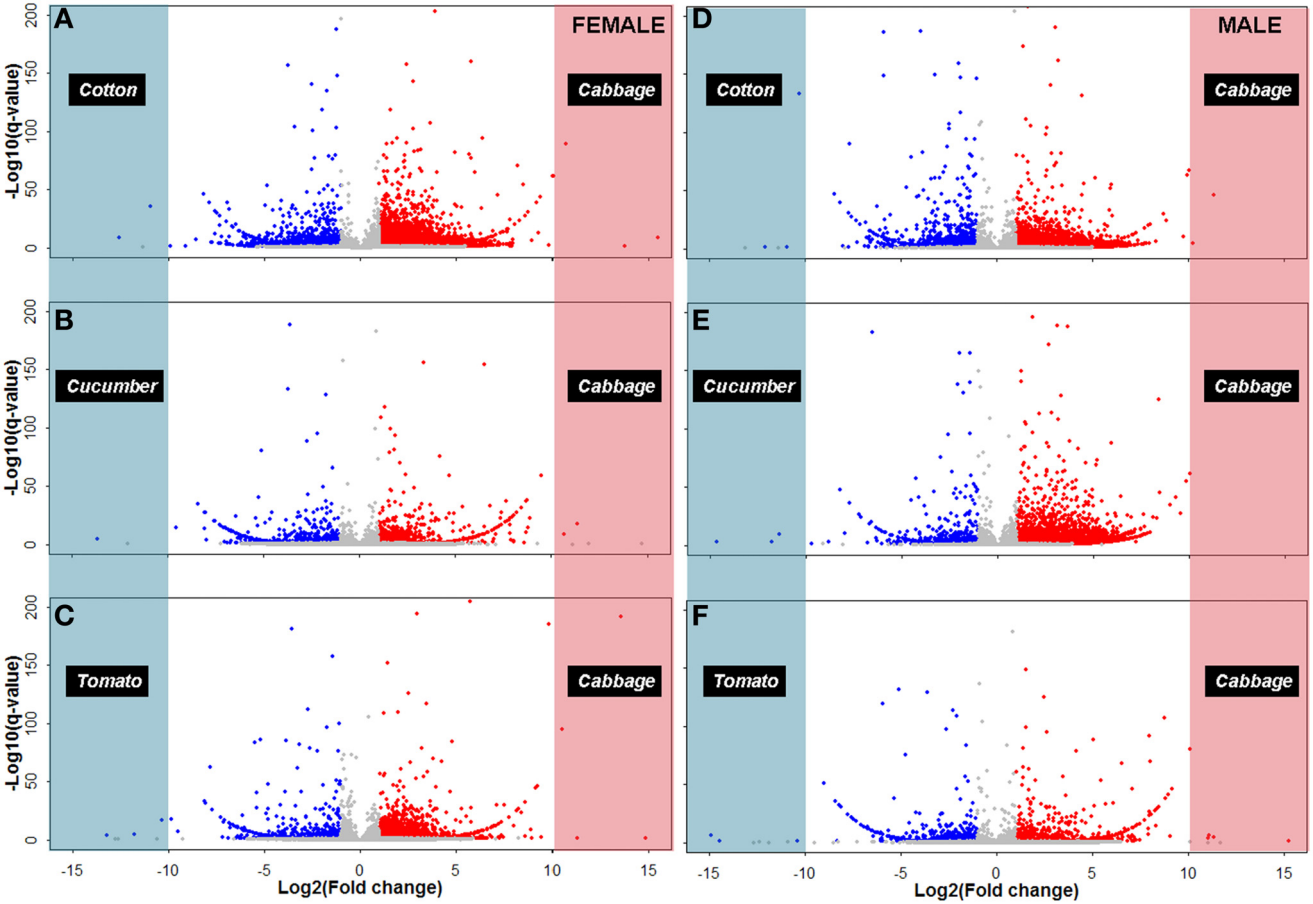
**Transcriptional differences between *B. tabaci* adults feeding on the original and the derived hosts**. The fold changes on x-axis represent the ratio of transcript abundance of *B. tabaci* adults on cabbage, cotton, cucumber and tomato host, respectively. Differentially expressed transcripts are highlighted in red [*q* < 0.05, log_2_ (fold change) >1] and blue [*q* < 0.05, log_2_ (fold change) < -1], respectively on the Volcano plot. **(A)** Cabbage female vs. cotton female; **(B)** Cabbage female vs. cucumber female; **(C)** Cabbage female vs. tomato female; **(D)** Cabbage male vs. cotton male; **(E)** Cabbage male vs. cucumber male; and **(F)** Cabbage male vs. tomato male.

To identify cabbage strain-biased genes, we compared gene expression profiles between Caf/Cof, Caf/Cuf, Caf/Tof, Cam/Com, Cam/Cum, and Cam/Tom, respectively. Differentially expressed transcripts (DETs) with *p* < 0.05 and log_2_ (Fold change) >1 or log_2_ (Fold change) <−1] were identified bioinformatically among each original/derived host pairings. In summary, 6294 up-regulated and 1139 down-regulated transcripts were found in Caf/Cof, 916 and 593 in Caf/Cuf, 2104 and 577 in Caf/Tof, 4257 and 1070 in Cam/Com, 5055 and 725 in Cam/Cum, 749 and 465 in Cam/Tom (Figures 2, 3; Figure S1; Table 2). Remarkably, in comparison to the derived hosts, 418 female and 303 male transcripts, respectively, were up-regulated in the original cabbage strain (Tables S1, S2). Among them, 17 transcripts were consistently up-regulated in both female and male whiteflies originated from the cabbage host (Table S9). Annotated transcripts (10) were subjected to the quantitative real-time PCR analysis (Tables S10, S11), in which 50% of the genes (5/10) were consistent with the RNAseq results. Specifically, two ESTs annotated as *Cathepsin B* or *Cathepsin B-like* genes were significantly up-regulated in the cabbage strain in comparison to the other three derive host strains (*P* < 0.01).

**Table 2:** **Differentially expressed genes between the original host and the derived hosts**.

**Host pairs**	**Differential expression genes**	Up-regulated^*^	Down-regulated^**^
Caf/Cof	7433	6294	1139
Caf/Cuf	1509	916	593
Caf/Tof	2681	2104	577
Cam/Com	5327	4257	1070
Cam/Cum	5780	5055	725
Cam/Tom	1214	749	465

* *Q -value < 0.05 and log_2_ (Fold change) >1*.

** *Q -value < 0.05 and log_2_ (Fold change) <-1*.

Cathepsin B and Cathepsin B-like cysteine protease genes represent large multigene families. Due to an unique “occluding loop,” these cysteine proteases can block the access of substrates and inhibitors in human (Musil et al., 1991; Illy et al., 1997). Recently, these cysteine proteases were found to play a key role in the biology of trematodes and to be important virulence factors in a picomplexan parasite *Eimeria tenella* (Rieux et al., 2012), liver fluke parasites *Fasciola hepatica* (Beckham et al., 2009) and *Fasciola gigantica* (Siricoon et al., 2012; Chantree et al., 2013). Previous study also shown that cowpea bruchids could fend off plant defensive efforts, such as dietary soybean cysteine protease inhibitor (scN), by over-expression of major digestive cathepsin L-like proteases as well as by activating scN-insensitive cathepsin B-like proteases (Moon et al., 2004; Koo et al., 2008; Jongsma and Beekwilder, 2011). When *B. tabaci* faces dietary challenges, such as host shift, Cathepsin B and Cathepsin B-like enzymes may play a similar role to compromise the chemical attack initiated by the host plants. Over-expression of these genes represents a transcriptomic response in whiteflies to xenobiotic compounds in cabbage phloem. Future studies involving cloning and functional characterization of these *Cathepsin B* and *Cathepsin B-like* genes are warranted to elucidate their involvement in the *B. tabaci* host adaptation mechanism.

## Conclusions

HAD, one of the driving forces for the speciation and diversification, has fascinated scientists for decades. In this study, host induced transcriptomic responses were investigated using four sweetpotato whitefly strains derived from a single cabbage host. *Bemisia tabaci*, a global invasive agricultural pest, has been notorious for its adaptation capability to different host plants and rapid range expansion/invasion for the past 50 years. Comparative transcriptomic analyses exhibited low level of transcriptional divergences between whiteflies maintained on the original host, cabbage, and the three derived hosts, cotton, cucumber and tomato. Although *B. tabaci* exhibits strong host plasticity in the field, this investigation suggests that the impact of HAD in whiteflies is limited at the transcription level. Therefore, host-associated adaptation is unlikely the primary factor contributing to the rapid range expansion/invasiveness in whiteflies.

## Author contributions

Conceived and designed the experiments: Wen Xie, Xuguo Zhou, Youjun Zhang. Performed the experiments: Wen Xie. Analyzed the data: Wen Xie, Xuguo Zhou. Contributed reagents/materials/analysis tools: Wen Xie, Qingjun Wu, Shaoli Wang, Xuguo Zhou, Youjun Zhang. Wrote the paper: Wen Xie, Qingjun Wu, Shaoli Wang, Xiaoguo Jiao Litao Guo, Xuguo Zhou, Youjun Zhang.

### Conflict of interest statement

The authors declare that the research was conducted in the absence of any commercial or financial relationships that could be construed as a potential conflict of interest.
