# Supplementary material for: Transcriptome analysis of host-associated differentiation in Bemisia tabaci (Hemiptera: Aleyrodidae)
Source: Front Physiol. 2014 Dec 10;5:487. doi: 10.3389/fphys.2014.00487 (PMC4261700; doi:10.3389/fphys.2014.00487)
Supplement: Supplementary file 1 [file DataSheet1.DOCX]

**Supplementary materials**

**Figure S1** **| Venn diagrams for down-regulated genes in host cabbage adult compared to other host adults.** **(A)** Down-regulated genes in cabbage female adult compared to cotton, cucumber and tomato female adults. Caf/Cof, down-regulated genes in cabbage female adult relative to that in cotton female adult. Caf/Tof, down-regulated genes in cabbage female adult relative to that in tomato female adult. Caf/Cuf, down-regulated genes in cabbage female adult relative to that in cucumber female adult. **(B)** Down-regulated genes in cabbage male adult compared to cotton, cucumber and tomato male adults. Caf/Cof, down-regulated genes in cabbage male adult relative to that in cotton male adult. Caf/Tof, down-regulated genes in cabbage male adult relative to that in tomato male adult. Caf/Cuf, down-regulated genes in cabbage male adult relative to that in cucumber male adult.

**Table S1 |** Annotation of cabbage female bias genes (Caf up-regulated) relative to that of other host female.

**Table S2 |** Annotation of cabbage male bias genes (Cam up-regulated) relative to that of other host male.

**Table S3 |** List of genes with Ka/Ks larger than one in Caf/Cof.

**Table S4 |** List of genes with Ka/Ks larger than one in Caf/Cuf.

**Table S5 |** List of genes with Ka/Ks larger than one in Caf/Tof.

**Table S6 |** List of genes with Ka/Ks larger than one in Cam/Com.

**Table S7 |** List of genes with Ka/Ks larger than one in Cam/Cum.

**Table S8 |** List of genes with Ka/Ks larger than one in Cam/Tom.

**Table S9 |** Annotation and expression level in RNA sequencing of 17 up-regulated genes no matter in cabbage female or in male comparative to that of other host female or male, respectively.

**Table S10 |** Primers used for the qRT-PCR analyses.

**Table S11 |** Validation and quantification of 10 up-regulated gene transcripts levels in original cabbage host according to the ∆CT method using *RPL29* as the reference gene (±S.E).

**Figure S1** **|** Venn diagrams for down-regulated genes in host cabbage adult compared to other host adults. **(A)** Down-regulated genes in cabbage female adult compared to cotton, cucumber and tomato female adults. Caf/Cof, down-regulated genes in cabbage female adult relative to that in cotton female adult. Caf/Tof, down-regulated genes in cabbage female adult relative to that in tomato female adult. Caf/Cuf, down-regulated genes in cabbage female adult relative to that in cucumber female adult. **(B)** Down-regulated genes in cabbage male adult compared to cotton, cucumber and tomato male adults. Caf/Cof, down-regulated genes in cabbage male adult relative to that in cotton male adult. Caf/Tof, down-regulated genes in cabbage male adult relative to that in tomato male adult. Caf/Cuf, down-regulated genes in cabbage male adult relative to that in cucumber male adult.

**Table S1 |** Annotation of cabbage female bias genes (Caf up-regulated) relative to that of other host female.

| **Assembly ID** | **NR database** |
| --- | --- |
| comp21_c0_seq1 | cytochrome c oxidase subunit III [Bemisia tabaci] gi\|51944947\|gb\|AAU14203.1\| cytochrome oxidase subunit III [Bemisia tabaci] |
| comp4580_c0_seq1 | PREDICTED: tudor domain-containing protein 7-like [Apis mellifera] |
| comp3874_c0_seq5 |  |
| comp2779_c0_seq1 |  |
| comp2420_c0_seq4 |  |
| comp11735_c0_seq1 |  |
| comp4580_c0_seq2 | PREDICTED: tudor domain-containing protein 7-like [Apis mellifera] |
| comp3706_c0_seq4 | PREDICTED: ataxin-3-like isoform 1 [Bombus terrestris] |
| comp1102_c1_seq1 |  |
| comp8149_c0_seq1 |  |
| comp3302_c0_seq3 |  |
| comp2754_c0_seq3 | hypothetical protein TcasGA2_TC002139 [Tribolium castaneum] |
| comp3470_c0_seq11 |  |
| comp5014_c0_seq2 |  |
| comp1575_c0_seq4 |  |
| comp7868_c0_seq1 | PREDICTED: similar to ENSANGP00000029312 [Nasonia vitripennis] |
| comp154_c0_seq1 |  |
| comp2378_c0_seq4 | dihydrodipicolinate reductase [Rickettsia felis URRWXCal2] gi\|75536068\|sp\|Q4UKE7.1\|DAPB_RICFE RecName: Full=Dihydrodipicolinate reductase; Short=DHPR &gt;gi\|67005058\|gb\|AAY61984.1\| Dihydrodipicolinate reductase [Rickettsia felis URRWXCal2] |
| comp2451_c0_seq2 |  |
| comp2378_c0_seq8 |  |
| comp694_c0_seq1 | RecName: Full=V-type proton ATPase 16 kDa proteolipid subunit; Short=V-ATPase 16 kDa proteolipid subunit; AltName: Full=Vacuolar proton pump 16 kDa proteolipid subunit gi\|290956\|gb\|AAC37176.1\| H+-ATPase V-type subunit [Heliothis virescens] |
| comp5461_c0_seq1 |  |
| comp6683_c0_seq1 |  |
| comp82_c0_seq1 | NADH dehydrogenase subunit 2 [Bemisia tabaci] gi\|51944948\|gb\|AAU14204.1\| NADH dehydrogenase subunit II [Bemisia tabaci] |
| comp522_c0_seq2 | cathepsin B-like cysteine protease form 1 [Ixodes ricinus] |
| comp5241_c0_seq1 | PREDICTED: gastrula zinc finger protein 5-1-like [Xenopus (Silurana) tropicalis] |
| comp10209_c0_seq3 |  |
| comp10447_c0_seq1 |  |
| comp6270_c0_seq2 | BTB/POZ domain-containing protein 3 [Acromyrmex echinatior] |
| comp822_c0_seq1 | hypothetical protein TcasGA2_TC004472 [Tribolium castaneum] |
| comp505_c0_seq1 |  |
| comp3507_c0_seq1 | ribosomal protein L21 [Candidatus Portiera aleyrodidarum] |
| comp10362_c1_seq1 |  |
| comp5957_c0_seq1 | THO complex subunit, putative [Pediculus humanus corporis] gi\|212507522\|gb\|EEB11443.1\| THO complex subunit, putative [Pediculus humanus corporis] |
| comp3720_c0_seq7 |  |
| comp1527_c0_seq5 |  |
| comp10362_c0_seq1 |  |
| comp2402_c1_seq4 | Deoxynucleoside kinase [Caligus clemensi] |
| comp6730_c0_seq1 |  |
| comp7222_c0_seq1 | sugar transporter [Aedes aegypti] gi\|108872280\|gb\|EAT36505.1\| sugar transporter [Aedes aegypti] |
| comp13011_c0_seq1 | PREDICTED: tyrosine-protein kinase Abl-like [Acyrthosiphon pisum] |
| comp7360_c0_seq1 |  |
| comp13842_c0_seq1 |  |
| comp2054_c0_seq4 |  |
| comp11110_c0_seq1 | secreted phosphatidylethanolamine binding protein [Glossina morsitans morsitans] |
| comp9174_c0_seq5 | Craniofacial development protein 2 [Harpegnathos saltator] |
| comp2256_c0_seq3 | J domain-containing protein C21orf55, putative [Pediculus humanus corporis] gi\|212514899\|gb\|EEB17127.1\| J domain-containing protein C21orf55, putative [Pediculus humanus corporis] |
| comp9327_c0_seq1 |  |
| comp18083_c0_seq1 |  |
| comp6244_c0_seq5 | PREDICTED: similar to AGAP009992-PA [Tribolium castaneum] |
| comp322_c0_seq1 | PREDICTED: similar to putative cathepsin B-like proteinase [Tribolium castaneum] gi\|270012705\|gb\|EFA09153.1\| cathepsin B precursor [Tribolium castaneum] |
| comp17885_c0_seq1 |  |
| comp3275_c0_seq1 |  |
| comp3794_c0_seq1 |  |
| comp14705_c0_seq2 |  |
| comp778_c2_seq3 | cysteine proteinase inhibitor precursor [Manduca sexta] gi\|261336196\|dbj\|BAH59606.2\| cysteine proteinase inhibitor precursor [Manduca sexta] |
| comp522_c0_seq1 | Cathepsin B precursor [Lepeophtheirus salmonis] gi\|290561811\|gb\|ADD38303.1\| Cathepsin B [Lepeophtheirus salmonis] |
| comp1984_c0_seq2 | eukaryotic translation initiation factor 4 gamma, putative [Pediculus humanus corporis] gi\|212506693\|gb\|EEB10826.1\| eukaryotic translation initiation factor 4 gamma, putative [Pediculus humanus corporis] |
| comp6519_c0_seq11 |  |
| comp6657_c0_seq1 | PREDICTED: integrin alpha-PS1-like isoform 1 [Acyrthosiphon pisum] |
| comp8883_c0_seq1 | putative H3K9 methyltransferase [Cercopis vulnerata] |
| comp2027_c0_seq1 |  |
| comp9811_c0_seq1 |  |
| comp15601_c0_seq1 | PREDICTED: UPF0533 protein C5orf44 homolog [Bombus terrestris] |
| comp7201_c0_seq1 |  |
| comp7451_c0_seq2 | PREDICTED: cAMP-dependent protein kinase catalytic subunit-like [Acyrthosiphon pisum] |
| comp1708_c0_seq4 |  |
| comp6733_c0_seq1 |  |
| comp3178_c0_seq2 |  |
| comp8788_c0_seq1 | PREDICTED: Antigen KI-67-like [Saccoglossus kowalevskii] |
| comp19366_c0_seq1 |  |
| comp4541_c0_seq1 | AGAP007494-PA [Anopheles gambiae str. PEST] gi\|157020060\|gb\|EAA04642.4\| AGAP007494-PA [Anopheles gambiae str. PEST] |
| comp3470_c0_seq2 | PREDICTED: similar to calcineurin A [Nasonia vitripennis] |
| comp297_c1_seq5 |  |
| comp12912_c0_seq1 |  |
| comp4970_c1_seq3 | Type I inositol-1,4,5-trisphosphate 5-phosphatase [Camponotus floridanus] |
| comp4247_c0_seq1 | PREDICTED: syntenin-1-like [Apis mellifera] |
| comp7402_c0_seq3 |  |
| comp488_c0_seq1 |  |
| comp3534_c0_seq4 |  |
| comp1030_c0_seq1 |  |
| comp6119_c0_seq1 |  |
| comp12011_c0_seq1 | PREDICTED: ras-related protein Rap-2c-like [Acyrthosiphon pisum] |
| comp8177_c0_seq1 |  |
| comp7049_c0_seq2 |  |
| comp13111_c0_seq1 |  |
| comp129_c0_seq1 |  |
| comp1493_c0_seq5 |  |
| comp7191_c0_seq2 | Leucine-rich repeat-containing protein 57 [Camponotus floridanus] |
| comp8617_c0_seq1 | PREDICTED: 3-hydroxyisobutyryl-CoA hydrolase, mitochondrial-like isoform 2 [Bombus terrestris] |
| comp4815_c0_seq1 | PREDICTED: FACT complex subunit spt16-like [Bombus terrestris] |
| comp5960_c0_seq2 | PREDICTED: similar to fascin [Tribolium castaneum] gi\|270012518\|gb\|EFA08966.1\| hypothetical protein TcasGA2_TC006673 [Tribolium castaneum] |
| comp9661_c0_seq1 | PREDICTED: similar to phospholipase D3 [Tribolium castaneum] gi\|270000747\|gb\|EEZ97194.1\| hypothetical protein TcasGA2_TC004381 [Tribolium castaneum] |
| comp4103_c0_seq2 | Zinc transporter foi [Harpegnathos saltator] |
| comp10262_c0_seq3 |  |
| comp5570_c0_seq5 |  |
| comp10287_c0_seq1 |  |
| comp3525_c0_seq1 |  |
| comp7328_c0_seq1 | GI17298 [Drosophila mojavensis] gi\|193912849\|gb\|EDW11716.1\| GI17298 [Drosophila mojavensis] |
| comp7817_c0_seq2 | PREDICTED: alpha-tocopherol transfer protein-like [Apis mellifera] |
| comp14342_c0_seq2 | PREDICTED: anoctamin-1-like [Acyrthosiphon pisum] |
| comp1670_c0_seq1 | glutathione S-transferase [Blattella germanica] |
| comp16639_c0_seq1 | PREDICTED: uncharacterized protein C1orf112 homolog [Bombus terrestris] |
| comp1677_c0_seq7 | Prefoldin subunit 6 [Acromyrmex echinatior] |
| comp14787_c0_seq1 |  |
| comp9427_c0_seq3 |  |
| comp2733_c0_seq1 |  |
| comp4573_c0_seq31 | ankyrin repeat protein [Trichomonas vaginalis G3] gi\|121906691\|gb\|EAY11596.1\| ankyrin repeat protein, putative [Trichomonas vaginalis G3] |
| comp197_c1_seq2 | PREDICTED: protein alan shepard-like [Acyrthosiphon pisum] |
| comp246_c1_seq1 | hypothetical protein TcasGA2_TC010324 [Tribolium castaneum] |
| comp2316_c0_seq4 | hypothetical protein SINV_12257 [Solenopsis invicta] |
| comp4829_c0_seq2 | PREDICTED: similar to calcium/calmodulin dependent protein kinase I [Nasonia vitripennis] |
| comp358_c0_seq1 |  |
| comp6072_c0_seq1 | TPA_inf: cathepsin B [Myzus persicae] |
| comp3586_c0_seq1 | DNA polymerase III subunit alpha [Reinekea sp. MED297] gi\|88779048\|gb\|EAR10237.1\| DNA polymerase III subunit alpha [Reinekea sp. MED297] |
| comp6937_c0_seq1 |  |
| comp11630_c0_seq2 | PREDICTED: similar to CG18472 CG18472-PA [Tribolium castaneum] gi\|270006748\|gb\|EFA03196.1\| hypothetical protein TcasGA2_TC013116 [Tribolium castaneum] |
| comp12455_c0_seq1 | PREDICTED: hypothetical protein LOC100164562 [Acyrthosiphon pisum] |
| comp13223_c0_seq1 |  |
| comp7712_c0_seq1 | PREDICTED: ATP-dependent DNA helicase PIF1-like [Acyrthosiphon pisum] |
| comp15530_c0_seq2 | PREDICTED: probable ATP-dependent RNA helicase YTHDC2 [Apis mellifera] |
| comp10347_c0_seq1 |  |
| comp10566_c0_seq1 |  |
| comp5677_c0_seq1 |  |
| comp10086_c0_seq1 |  |
| comp7681_c0_seq3 |  |
| comp139_c2_seq4 | PREDICTED: mucolipin-3-like [Bombus terrestris] |
| comp1359_c0_seq1 |  |
| comp5425_c0_seq2 |  |
| comp6738_c0_seq2 |  |
| comp1595_c0_seq1 | Splicing factor, arginine/serine-rich 1 [Acromyrmex echinatior] |
| comp8212_c0_seq1 | hypothetical protein SINV_07700 [Solenopsis invicta] |
| comp10781_c0_seq1 |  |
| comp9184_c0_seq1 | aldehyde dehydrogenase [Leadbetterella byssophila DSM 17132] gi\|311905570\|gb\|ADQ16011.1\| Aldehyde Dehydrogenase [Leadbetterella byssophila DSM 17132] |
| comp1951_c0_seq2 |  |
| comp8927_c0_seq1 |  |
| comp3034_c0_seq6 |  |
| comp2219_c0_seq5 |  |
| comp12439_c0_seq1 |  |
| comp4189_c0_seq5 |  |
| comp10699_c0_seq2 | hypothetical protein TcasGA2_TC011089 [Tribolium castaneum] |
| comp13367_c0_seq1 | PREDICTED: hypothetical protein LOC413802 [Apis mellifera] |
| comp10633_c0_seq1 | PREDICTED: zinc finger protein 271-like [Macaca mulatta] |
| comp2515_c0_seq8 | PREDICTED: hypothetical protein LOC726033 [Apis mellifera] |
| comp75_c0_seq2 | hypothetical protein CAEBREN_32771 [Caenorhabditis brenneri] |
| comp8205_c0_seq1 |  |
| comp4093_c0_seq6 |  |
| comp10052_c0_seq1 |  |
| comp11643_c0_seq1 | PREDICTED: uncharacterized protein C45G9.7-like [Apis mellifera] gi\|340712148\|ref\|XP_003394626.1\| PREDICTED: uncharacterized protein C45G9.7-like [Bombus terrestris] |
| comp15693_c0_seq1 | hypothetical protein TcasGA2_TC003006 [Tribolium castaneum] |
| comp6918_c0_seq1 |  |
| comp8764_c0_seq1 | Coronin-7 [Harpegnathos saltator] |
| comp8388_c0_seq1 | PREDICTED: transcription factor E2F2-like isoform 1 [Acyrthosiphon pisum] gi\|328708305\|ref\|XP_003243650.1\| PREDICTED: transcription factor E2F2-like isoform 2 [Acyrthosiphon pisum] |
| comp938_c1_seq1 |  |
| comp968_c0_seq1 | Glycerol-3-phosphate acyltransferase 1, mitochondrial [Acromyrmex echinatior] |
| comp3015_c0_seq1 | hypothetical protein SINV_09125 [Solenopsis invicta] |
| comp5413_c0_seq1 | pre-mRNA-splicing factor ATP-dependent RNA helicase prp16, putative [Pediculus humanus corporis] gi\|212511335\|gb\|EEB14339.1\| pre-mRNA-splicing factor ATP-dependent RNA helicase prp16, putative [Pediculus humanus corporis] |
| comp16533_c0_seq1 | PREDICTED: protein OPI10 homolog [Apis mellifera] |
| comp16120_c0_seq1 | PREDICTED: hypothetical protein LOC100647007 [Bombus terrestris] |
| comp13843_c0_seq1 |  |
| comp10657_c0_seq1 |  |
| comp6908_c0_seq1 | hypothetical protein TcasGA2_TC012938 [Tribolium castaneum] |
| comp6412_c0_seq1 | hypothetical protein SINV_09204 [Solenopsis invicta] |
| comp8588_c0_seq2 |  |
| comp5204_c0_seq1 |  |
| comp6862_c0_seq1 | PREDICTED: 52 kDa repressor of the inhibitor of the protein kinase [Macaca mulatta] |
| comp14098_c0_seq2 | hypothetical protein SINV_00394 [Solenopsis invicta] |
| comp18709_c0_seq1 |  |
| comp4442_c0_seq1 | GTP-binding protein Rheb-like [Acyrthosiphon pisum] gi\|239791527\|dbj\|BAH72216.1\| ACYPI008618 [Acyrthosiphon pisum] |
| comp5934_c0_seq2 |  |
| comp2502_c0_seq1 |  |
| comp1644_c0_seq3 | ELAV-like protein 2 [Harpegnathos saltator] |
| comp5704_c0_seq2 |  |
| comp18_c0_seq1 | NADH dehydrogenase subunit 5 [Bemisia tabaci] gi\|51944941\|gb\|AAU14197.1\| NADH dehydrogenase subunit 5 [Bemisia tabaci] |
| comp9650_c0_seq1 |  |
| comp9116_c0_seq1 |  |
| comp10450_c0_seq2 | PREDICTED: dystrobrevin beta-like [Acyrthosiphon pisum] |
| comp2773_c0_seq1 | putative rab11 [Homalodisca vitripennis] |
| comp5574_c0_seq1 |  |
| comp5142_c0_seq1 | PREDICTED: similar to conserved hypothetical protein [Nasonia vitripennis] |
| comp1209_c0_seq2 |  |
| comp6167_c0_seq1 |  |
| comp1455_c0_seq1 |  |
| comp3781_c0_seq1 | RING finger and WD repeat domain protein, putative [Pediculus humanus corporis] gi\|212516326\|gb\|EEB18339.1\| RING finger and WD repeat domain protein, putative [Pediculus humanus corporis] |
| comp758_c1_seq2 | PREDICTED: facilitated trehalose transporter Tret1-like [Acyrthosiphon pisum] |
| comp5299_c0_seq1 | ACYPI009740 [Acyrthosiphon pisum] |
| comp2938_c0_seq4 | PREDICTED: hypothetical protein LOC100158735 [Acyrthosiphon pisum] |
| comp1016_c2_seq1 | predicted protein [Pediococcus acidilactici 7_4] gi\|270281388\|gb\|EFA27220.1\| predicted protein [Pediococcus acidilactici 7_4] |
| comp139_c2_seq3 |  |
| comp3470_c0_seq6 |  |
| comp7947_c0_seq1 | MYND domain protein, putative [Penicillium marneffei ATCC 18224] gi\|210068786\|gb\|EEA22877.1\| MYND domain protein, putative [Penicillium marneffei ATCC 18224] |
| comp2559_c0_seq5 |  |
| comp18456_c0_seq1 | PREDICTED: similar to histone-lysine n-methyltransferase [Nasonia vitripennis] |
| comp1608_c0_seq2 |  |
| comp11173_c0_seq1 |  |
| comp2365_c0_seq2 | peptide methionine sulfoxide reductase MsrA [Triatoma matogrossensis] |
| comp96_c1_seq1 | RER1 protein, putative [Pediculus humanus corporis] gi\|212515284\|gb\|EEB17450.1\| RER1 protein, putative [Pediculus humanus corporis] |
| comp5510_c0_seq1 | PREDICTED: nogo-B receptor-like [Acyrthosiphon pisum] gi\|239790664\|dbj\|BAH71880.1\| ACYPI27127 [Acyrthosiphon pisum] |
| comp2565_c0_seq1 |  |
| comp4991_c0_seq1 |  |
| comp4646_c0_seq1 | PREDICTED: similar to predicted protein [Tribolium castaneum] |
| comp11978_c0_seq1 | PREDICTED: cyclin-Y-like [Acyrthosiphon pisum] |
| comp9389_c1_seq1 | PREDICTED: similar to MGC79091 protein [Tribolium castaneum] gi\|270009431\|gb\|EFA05879.1\| hypothetical protein TcasGA2_TC008691 [Tribolium castaneum] |
| comp6010_c0_seq1 | PREDICTED: septin-1-like [Apis mellifera] |
| comp6471_c0_seq3 |  |
| comp11876_c0_seq1 |  |
| comp4733_c0_seq1 | PREDICTED: amyloid beta A4 precursor protein-binding family B member 1-interacting protein-like [Acyrthosiphon pisum] gi\|328720547\|ref\|XP_003247064.1\| PREDICTED: amyloid beta A4 precursor protein-binding family B member 1-interacting protein-like [Acyrthosiphon pisum] |
| comp11245_c0_seq1 |  |
| comp12157_c0_seq1 |  |
| comp1368_c0_seq1 |  |
| comp5753_c0_seq2 |  |
| comp687_c0_seq2 | hypothetical protein TcasGA2_TC012700 [Tribolium castaneum] |
| comp231_c0_seq1 | squid, variant G [Blattella germanica] |
| comp18760_c0_seq1 | PREDICTED: hypothetical protein LOC100568486 [Acyrthosiphon pisum] |
| comp217_c0_seq3 |  |
| comp15457_c0_seq3 | PREDICTED: origin recognition complex subunit 2-like [Acyrthosiphon pisum] |
| comp5967_c0_seq1 | hypothetical protein TcasGA2_TC003450 [Tribolium castaneum] |
| comp6216_c0_seq1 | hypothetical protein TcasGA2_TC011468 [Tribolium castaneum] |
| comp4500_c0_seq1 | PREDICTED: probable maltase H [Acyrthosiphon pisum] |
| comp5469_c0_seq2 |  |
| comp3832_c0_seq2 |  |
| comp7933_c0_seq1 |  |
| comp8898_c0_seq1 |  |
| comp1215_c0_seq1 | PREDICTED: hypothetical protein LOC100164801 isoform 2 [Acyrthosiphon pisum] |
| comp6858_c0_seq1 |  |
| comp226_c0_seq1 |  |
| comp13916_c0_seq2 | E3 ubiquitin-protein ligase RAD18 [Harpegnathos saltator] |
| comp12301_c0_seq1 |  |
| comp13505_c0_seq1 | PREDICTED: DNA mismatch repair protein Mlh1-like [Acyrthosiphon pisum] |
| comp1474_c1_seq8 | hypothetical protein AND_07144 [Anopheles darlingi] |
| comp9312_c0_seq1 |  |
| comp6165_c0_seq3 |  |
| comp3509_c0_seq1 | PREDICTED: LOW QUALITY PROTEIN: ribosomal protein S6 kinase beta-1-like [Bombus terrestris] |
| comp71_c0_seq1 |  |
| comp12235_c0_seq1 | glutathione-S-transferase theta, GST, putative [Pediculus humanus corporis] gi\|212510153\|gb\|EEB13368.1\| glutathione-S-transferase theta, GST, putative [Pediculus humanus corporis] |
| comp2272_c0_seq1 | hypothetical protein TcasGA2_TC013386 [Tribolium castaneum] |
| comp2360_c0_seq1 | PREDICTED: similar to ubiquitin conjugating enzyme-like protein [Tribolium castaneum] gi\|270004126\|gb\|EFA00574.1\| hypothetical protein TcasGA2_TC003444 [Tribolium castaneum] |
| comp5566_c0_seq1 | hypothetical protein SINV_80284 [Solenopsis invicta] |
| comp4993_c0_seq1 | PREDICTED: protein scalloped-like [Bombus terrestris] |
| comp7207_c0_seq1 |  |
| comp5207_c0_seq1 | PREDICTED: similar to predicted protein [Tribolium castaneum] |
| comp380_c2_seq7 |  |
| comp4125_c0_seq6 | synoviolin, putative [Pediculus humanus corporis] gi\|212514520\|gb\|EEB16828.1\| synoviolin, putative [Pediculus humanus corporis] |
| comp4020_c0_seq1 |  |
| comp9385_c0_seq1 | PREDICTED: coiled-coil domain-containing protein 12-like isoform 1 [Apis mellifera] |
| comp1543_c0_seq1 |  |
| comp6852_c0_seq1 | Amyloid protein-binding protein 2 [Harpegnathos saltator] |
| comp2667_c0_seq2 | PREDICTED: similar to C20orf18 [Strongylocentrotus purpuratus] gi\|115974996\|ref\|XP_001187511.1\| PREDICTED: similar to C20orf18 [Strongylocentrotus purpuratus] |
| comp4032_c0_seq1 | PREDICTED: hypothetical protein LOC411348 [Apis mellifera] |
| comp1853_c0_seq1 | PREDICTED: e3 ubiquitin-protein ligase KCMF1-like [Apis mellifera] |
| comp10905_c0_seq2 | Mitogen-activated protein kinase kinase kinase kinase 5 [Harpegnathos saltator] |
| comp12138_c0_seq1 | FL(2)D protein, putative [Glyptapanteles indiensis] |
| comp9438_c0_seq2 |  |
| comp7320_c0_seq1 |  |
| comp3691_c0_seq1 | PREDICTED: v-type proton ATPase 116 kDa subunit a isoform 1-like [Acyrthosiphon pisum] |
| comp10097_c0_seq1 | protein angel [Acyrthosiphon pisum] gi\|328726434\|ref\|XP_003248895.1\| PREDICTED: LOW QUALITY PROTEIN: protein angel homolog 2-like [Acyrthosiphon pisum] |
| comp3431_c0_seq2 | bhlhzip transcription factor max/bigmax-like [Acyrthosiphon pisum] gi\|239790453\|dbj\|BAH71787.1\| ACYPI005523 [Acyrthosiphon pisum] |
| comp1935_c0_seq2 |  |
| comp5658_c0_seq1 | beta-glucosidase [Odontotermes formosanus] |
| comp1658_c0_seq1 |  |
| comp8423_c0_seq3 |  |
| comp5077_c0_seq1 | hypothetical protein DAPPUDRAFT_95812 [Daphnia pulex] |
| comp75_c0_seq1 | hypothetical protein CAEBREN_32771 [Caenorhabditis brenneri] |
| comp2135_c0_seq1 |  |
| comp7792_c0_seq1 |  |
| comp9883_c0_seq1 |  |
| comp8519_c0_seq1 |  |
| comp9213_c0_seq1 | PREDICTED: AP-1 complex subunit gamma-1-like isoform 2 [Bombus terrestris] |
| comp9711_c0_seq1 | PREDICTED: inositol-tetrakisphosphate 1-kinase-like [Acyrthosiphon pisum] |
| comp7659_c0_seq1 |  |
| comp9407_c0_seq1 | Histone-lysine N-methyltransferase Suv4-20 [Acromyrmex echinatior] |
| comp5004_c0_seq1 | PREDICTED: trafficking protein particle complex subunit 3-like [Bombus terrestris] |
| comp11965_c0_seq3 |  |
| comp6140_c0_seq6 |  |
| comp1396_c0_seq1 | cyclopropane fatty acyl phospholipid synthase [Cronobacter sakazakii ATCC BAA-894] gi\|156532449\|gb\|ABU77275.1\| hypothetical protein ESA_02022 [Cronobacter sakazakii ATCC BAA-894] |
| comp4062_c0_seq1 |  |
| comp1539_c0_seq1 | PREDICTED: microtubule-associated protein RP/EB family member 3-like isoform 2 [Acyrthosiphon pisum] |
| comp15255_c0_seq1 | conserved hypothetical protein [Culex quinquefasciatus] gi\|167864147\|gb\|EDS27530.1\| conserved hypothetical protein [Culex quinquefasciatus] |
| comp13629_c0_seq1 |  |
| comp9079_c0_seq1 | PREDICTED: hypothetical protein LOC100646252 [Bombus terrestris] |
| comp13054_c0_seq1 | PREDICTED: hypothetical protein LOC100642825 isoform 2 [Bombus terrestris] |
| comp6248_c0_seq1 | PREDICTED: endoplasmic reticulum aminopeptidase 2-like [Acyrthosiphon pisum] |
| comp9177_c0_seq1 | protein C kinase 98E-like protein [Tribolium castaneum] |
| comp1831_c0_seq1 | PREDICTED: hypothetical protein LOC100164977 [Acyrthosiphon pisum] |
| comp6271_c0_seq1 |  |
| comp6818_c0_seq1 | Cytoplasmic protein NCK1, putative [Pediculus humanus corporis] gi\|212517869\|gb\|EEB19698.1\| Cytoplasmic protein NCK1, putative [Pediculus humanus corporis] |
| comp3056_c0_seq1 | Uncharacterized protein C9orf85-like protein [Harpegnathos saltator] |
| comp311_c0_seq1 | Phosphoglycerate mutase 1 [Acromyrmex echinatior] |
| comp747_c0_seq2 | PREDICTED: similar to ENSANGP00000007272 [Nasonia vitripennis] |
| comp11216_c0_seq1 | PREDICTED: integrator complex subunit 8-like [Bombus terrestris] |
| comp7507_c0_seq1 | leucine-rich repeat protein [Rickettsia endosymbiont of Ixodes scapularis] gi\|239922315\|gb\|EER22339.1\| leucine-rich repeat protein [Rickettsia endosymbiont of Ixodes scapularis] |
| comp3419_c0_seq1 | PREDICTED: glycerol-3-phosphate acyltransferase 4-like [Acyrthosiphon pisum] |
| comp9966_c0_seq2 | PREDICTED: UPF0459 protein CG10681-like [Apis mellifera] |
| comp2632_c1_seq1 |  |
| comp4974_c0_seq4 | PREDICTED: similar to conserved hypothetical protein [Nasonia vitripennis] |
| comp663_c0_seq1 |  |
| comp6914_c0_seq1 |  |
| comp427_c0_seq1 | NADH:ubiquinone reductase 42kD subunit precursor, isoform A [Drosophila melanogaster] gi\|24648886\|ref\|NP_732692.1\| NADH:ubiquinone reductase 42kD subunit precursor, isoform B [Drosophila melanogaster] &gt;gi\|12644364\|sp\|P91929.2\|NDUAA_DROME RecName: Full=NADH dehydrogenase [ubiquinone] 1 alpha subcomplex subunit 10, mitochondrial; AltName: Full=Complex I-42kD; Short=CI-42kD; AltName: Full=NADH-ubiquinone oxidoreductase 42 kDa subunit; Flags: Precursor &gt;gi\|7300804\|gb\|AAF55947.1\| NADH:ubiquinone reductase 42kD subunit precursor, isoform A [Drosophila melanogaster] &gt;gi\|15291839\|gb\|AAK93188.1\| LD29280p [Drosophila melanogaster] &gt;gi\|23171948\|gb\|AAN13899.1\| NADH:ubiquinone reductase 42kD subunit precursor, isoform B [Drosophila melanogaster] &gt;gi\|220945916\|gb\|ACL85501.1\| ND42-PA [synthetic construct] &gt;gi\|220955674\|gb\|ACL90380.1\| ND42-PA [synthetic construct] |
| comp3093_c0_seq2 | GTP-binding protein 128up, putative [Pediculus humanus corporis] gi\|212517682\|gb\|EEB19535.1\| GTP-binding protein 128up, putative [Pediculus humanus corporis] |
| comp6644_c0_seq3 | PREDICTED: actin-like protein 6A-like isoform 1 [Bombus terrestris] gi\|340724324\|ref\|XP_003400532.1\| PREDICTED: actin-like protein 6A-like isoform 2 [Bombus terrestris] |
| comp2196_c0_seq1 | PREDICTED: chromosome-associated kinesin KIF4-like [Acyrthosiphon pisum] |
| comp2527_c0_seq2 | PREDICTED: ATP-binding cassette sub-family G member 4-like isoform 2 [Acyrthosiphon pisum] |
| comp3002_c0_seq1 | PREDICTED: kinesin-like protein KIF2A-like isoform 1 [Acyrthosiphon pisum] |
| comp641_c0_seq3 |  |
| comp15186_c0_seq1 | PREDICTED: 2-aminoethanethiol dioxygenase-like [Acyrthosiphon pisum] |
| comp1495_c1_seq2 | Casein kinase II subunit alpha [Harpegnathos saltator] |
| comp1097_c0_seq4 |  |
| comp12182_c0_seq2 | PREDICTED: serine/threonine-protein kinase PAK 7-like [Acyrthosiphon pisum] |
| comp8812_c0_seq1 |  |
| comp6284_c0_seq1 | Exportin-2 [Harpegnathos saltator] |
| comp7889_c0_seq1 |  |
| comp2750_c0_seq1 | PREDICTED: plastin-3-like [Acyrthosiphon pisum] |
| comp540_c0_seq4 |  |
| comp158_c0_seq1 | PREDICTED: SPRY domain-containing SOCS box protein 3-like [Apis mellifera] |
| comp3213_c1_seq2 |  |
| comp7860_c0_seq1 | PREDICTED: similar to CG9008 CG9008-PA [Tribolium castaneum] |
| comp6859_c0_seq1 |  |
| comp1209_c0_seq1 |  |
| comp13342_c0_seq1 |  |
| comp528_c4_seq1 | predicted protein [Hordeum vulgare subsp. vulgare] |
| comp2175_c0_seq1 | hypothetical protein DAPPUDRAFT_310616 [Daphnia pulex] |
| comp5810_c0_seq1 | inositol monophosphatase family domain containing protein [Pediculus humanus corporis] gi\|212513972\|gb\|EEB16370.1\| inositol monophosphatase family domain containing protein [Pediculus humanus corporis] |
| comp4936_c0_seq1 | hypothetical protein TcasGA2_TC002424 [Tribolium castaneum] |
| comp5952_c0_seq3 | PREDICTED: ubiquitin carboxyl-terminal hydrolase 8-like [Acyrthosiphon pisum] |
| comp2019_c0_seq1 | PREDICTED: similar to ENSANGP00000011247 [Nasonia vitripennis] |
| comp12030_c0_seq2 | GK11189 [Drosophila willistoni] gi\|194166224\|gb\|EDW81125.1\| GK11189 [Drosophila willistoni] |
| comp7594_c0_seq4 |  |
| comp2828_c0_seq4 | extracellular regulated MAP kinase [Bombyx mori] gi\|77799292\|dbj\|BAE46741.1\| Extracellular regulated MAP kinase [Bombyx mori] |
| comp5930_c0_seq4 | Cylicin-1, putative [Pediculus humanus corporis] gi\|212517483\|gb\|EEB19371.1\| Cylicin-1, putative [Pediculus humanus corporis] |
| comp1184_c0_seq4 | PREDICTED: hypothetical protein LOC100648913 [Bombus terrestris] |
| comp10811_c0_seq1 | Gb1-cadherin [Gryllus bimaculatus] |
| comp12142_c0_seq1 | PREDICTED: rab GTPase-activating protein 1-like isoform 2 [Bombus terrestris] |
| comp5281_c0_seq1 | PREDICTED: hypothetical protein LOC100164619 [Acyrthosiphon pisum] |
| comp11231_c0_seq2 |  |
| comp7272_c0_seq1 | hypothetical protein TcasGA2_TC009925 [Tribolium castaneum] |
| comp7527_c0_seq1 |  |
| comp5131_c0_seq1 | calcineurin B-like [Acyrthosiphon pisum] gi\|239790671\|dbj\|BAH71883.1\| ACYPI003710 [Acyrthosiphon pisum] |
| comp6878_c0_seq1 | hypothetical protein TcasGA2_TC014713 [Tribolium castaneum] |
| comp10389_c0_seq1 |  |
| comp5577_c0_seq1 | PREDICTED: PMS1 protein homolog 1-like [Acyrthosiphon pisum] |
| comp14206_c0_seq1 | PREDICTED: origin recognition complex subunit 3-like [Acyrthosiphon pisum] |
| comp12202_c0_seq1 | KLRAQ motif-containing protein 1 [Xenopus laevis] gi\|82185086\|sp\|Q6IR70.1\|KLRAQ_XENLA RecName: Full=KLRAQ motif-containing protein 1; AltName: Full=Coiled-coil domain-containing protein 128 &gt;gi\|47507513\|gb\|AAH71030.1\| MGC82266 protein [Xenopus laevis] |
| comp6293_c0_seq1 | PEST proteolytic signal-containing nuclear protein, putative [Pediculus humanus corporis] gi\|212514560\|gb\|EEB16855.1\| PEST proteolytic signal-containing nuclear protein, putative [Pediculus humanus corporis] |
| comp9237_c0_seq1 |  |
| comp516_c0_seq2 |  |
| comp8218_c0_seq1 | conserved hypothetical protein [Pediculus humanus corporis] gi\|212506988\|gb\|EEB11034.1\| conserved hypothetical protein [Pediculus humanus corporis] |
| comp1897_c0_seq2 |  |
| comp6722_c0_seq3 | PREDICTED: similar to LOC496249 protein [Ciona intestinalis] |
| comp2674_c0_seq2 |  |
| comp3706_c0_seq2 | PREDICTED: similar to phosphoinositide-binding protein, putative [Tribolium castaneum] gi\|270006674\|gb\|EFA03122.1\| hypothetical protein TcasGA2_TC013032 [Tribolium castaneum] |
| comp2879_c0_seq3 | PREDICTED: la-related protein 4-like [Apis mellifera] |
| comp4285_c0_seq6 | PREDICTED: similar to sorting nexin isoform 1 [Tribolium castaneum] gi\|270009518\|gb\|EFA05966.1\| hypothetical protein TcasGA2_TC008785 [Tribolium castaneum] |
| comp13421_c0_seq1 | hypothetical protein TcasGA2_TC012416 [Tribolium castaneum] |
| comp1896_c0_seq1 | Stathmin-4 [Acromyrmex echinatior] |
| comp7849_c0_seq1 | PREDICTED: similar to ribosome biogenesis protein brix [Nasonia vitripennis] |
| comp9369_c0_seq1 | PREDICTED: Werner syndrome ATP-dependent helicase homolog [Acyrthosiphon pisum] |
| comp5310_c0_seq2 |  |
| comp12310_c0_seq1 | PREDICTED: dual specificity mitogen-activated protein kinase kinase 7-like [Acyrthosiphon pisum] |
| comp4666_c0_seq1 |  |
| comp4759_c0_seq3 | RecName: Full=Nitric oxide synthase, salivary gland; Short=NOS gi\|1418270\|gb\|AAB03810.1\| nitric oxide synthase [Rhodnius prolixus] |
| comp8041_c0_seq1 | PREDICTED: similar to conserved hypothetical protein, partial [Nasonia vitripennis] |
| comp7587_c0_seq3 | tyrosine-protein kinase transmembrane receptor ROR1 precursor, putative [Pediculus humanus corporis] gi\|212515996\|gb\|EEB18061.1\| tyrosine-protein kinase transmembrane receptor ROR1 precursor, putative [Pediculus humanus corporis] |
| comp11430_c0_seq2 | protein phosphatase 1 regulatory subunit, putative [Pediculus humanus corporis] gi\|212510746\|gb\|EEB13859.1\| protein phosphatase 1 regulatory subunit, putative [Pediculus humanus corporis] |
| comp6074_c0_seq1 | hypothetical protein AaeL_AAEL010091 [Aedes aegypti] gi\|108873750\|gb\|EAT37975.1\| conserved hypothetical protein [Aedes aegypti] |
| comp2291_c0_seq1 | PREDICTED: similar to ENSANGP00000020237 [Nasonia vitripennis] |
| comp5901_c0_seq1 | PREDICTED: hypothetical protein [Nasonia vitripennis] |
| comp2338_c0_seq1 | PREDICTED: phosphatidylinositol N-acetylglucosaminyltransferase subunit C-like [Acyrthosiphon pisum] |
| comp5888_c0_seq2 | hypothetical protein DAPPUDRAFT_307206 [Daphnia pulex] |
| comp5622_c0_seq3 | DEAD-box helicase Dbp80 [Harpegnathos saltator] |
| comp7962_c0_seq1 | hypothetical protein TcasGA2_TC004421 [Tribolium castaneum] |
| comp0_c0_seq1 |  |
| comp7727_c0_seq2 | PREDICTED: hypothetical protein LOC100572526 [Acyrthosiphon pisum] |
| comp4791_c0_seq1 | PREDICTED: similar to GA19694-PA [Nasonia vitripennis] |
| comp4522_c0_seq1 |  |
| comp8598_c0_seq2 | RING finger protein, putative [Pediculus humanus corporis] gi\|212510757\|gb\|EEB13870.1\| RING finger protein, putative [Pediculus humanus corporis] |
| comp5164_c0_seq1 | PREDICTED: similar to srpk [Nasonia vitripennis] |
| comp2461_c0_seq1 | hypothetical protein TcasGA2_TC003916 [Tribolium castaneum] |
| comp6979_c0_seq1 | PREDICTED: hypothetical protein LOC100642720 [Bombus terrestris] |
| comp3142_c0_seq2 | PREDICTED: KH domain-containing, RNA-binding, signal transduction-associated protein 3-like isoform 1 [Apis mellifera] |
| comp2845_c0_seq2 | PREDICTED: ATP-binding cassette sub-family B member 10, mitochondrial-like [Acyrthosiphon pisum] |
| comp1625_c0_seq1 |  |
| comp9563_c0_seq1 | PREDICTED: similar to 5-3 exoribonuclease 1 [Nasonia vitripennis] |
| comp6765_c0_seq1 | PREDICTED: similar to predicted protein [Tribolium castaneum] gi\|270000885\|gb\|EEZ97332.1\| hypothetical protein TcasGA2_TC011144 [Tribolium castaneum] |
| comp3127_c0_seq2 | Tau-tubulin kinase 1 [Harpegnathos saltator] |
| comp12579_c0_seq1 | PREDICTED: hypothetical protein LOC100650463 [Bombus terrestris] |
| comp4567_c0_seq2 | Abhydrolase domain-containing protein 4 [Acromyrmex echinatior] |
| comp4390_c0_seq1 | PREDICTED: similar to rCG61344 [Nasonia vitripennis] |
| comp4271_c0_seq1 | PREDICTED: similar to ENSANGP00000010004 [Nasonia vitripennis] |
| comp3789_c1_seq1 | PREDICTED: similar to ras-related protein Rab-8A, putative [Tribolium castaneum] |
| comp5415_c0_seq2 | PREDICTED: prolyl 4-hydroxylase subunit alpha-2-like isoform 2 [Acyrthosiphon pisum] |
| comp1427_c0_seq1 | Guanine nucleotide-binding protein gamma-1 subunit precursor, putative [Pediculus humanus corporis] gi\|212511804\|gb\|EEB14686.1\| Guanine nucleotide-binding protein gamma-1 subunit precursor, putative [Pediculus humanus corporis] |
| comp5058_c0_seq1 | PREDICTED: e3 ubiquitin ligase RNF4-like [Monodelphis domestica] |
| comp1702_c0_seq1 | PREDICTED: similar to AGAP009119-PA [Tribolium castaneum] gi\|270014189\|gb\|EFA10637.1\| hypothetical protein TcasGA2_TC016274 [Tribolium castaneum] |
| comp6285_c0_seq1 | PREDICTED: similar to conserved hypothetical protein [Nasonia vitripennis] |
| comp7549_c0_seq1 |  |
| comp6512_c0_seq1 | PREDICTED: transcription termination factor 2-like isoform 1 [Acyrthosiphon pisum] gi\|328713458\|ref\|XP_003245082.1\| PREDICTED: transcription termination factor 2-like isoform 2 [Acyrthosiphon pisum] |
| comp9732_c0_seq1 |  |
| comp5984_c0_seq1 | hypothetical protein Phum_PHUM472670 [Pediculus humanus corporis] gi\|212515426\|gb\|EEB17573.1\| hypothetical protein Phum_PHUM472670 [Pediculus humanus corporis] |
| comp7051_c0_seq1 | hypothetical protein AaeL_AAEL002924 [Aedes aegypti] gi\|108881602\|gb\|EAT45827.1\| conserved hypothetical protein [Aedes aegypti] |
| comp1357_c0_seq1 |  |
| comp1784_c0_seq3 | PREDICTED: catenin delta-2-like isoform 1 [Acyrthosiphon pisum] |
| comp8491_c0_seq1 |  |
| comp3873_c0_seq1 | PREDICTED: uncharacterized protein DKFZp781G0119-like [Mus musculus] |
| comp5175_c0_seq3 | conserved hypothetical protein [Pediculus humanus corporis] gi\|212508158\|gb\|EEB11927.1\| conserved hypothetical protein [Pediculus humanus corporis] |
| comp5918_c0_seq1 | PREDICTED: prostatic acid phosphatase-like [Bombus terrestris] |
| comp1538_c0_seq2 | RAB GDP/GTP exchange factor, putative [Pediculus humanus corporis] gi\|212511201\|gb\|EEB14234.1\| RAB GDP/GTP exchange factor, putative [Pediculus humanus corporis] |
| comp337_c0_seq1 | PREDICTED: similar to Haspin CG40080-PA [Tribolium castaneum] |
| comp5792_c0_seq1 | Muskelin, putative [Pediculus humanus corporis] gi\|212513118\|gb\|EEB15746.1\| Muskelin, putative [Pediculus humanus corporis] |
| comp1828_c0_seq1 | PREDICTED: hypothetical protein LOC100639010 [Amphimedon queenslandica] |
| comp4532_c0_seq1 | AGAP008118-PA [Anopheles gambiae str. PEST] gi\|157015001\|gb\|EAA12568.4\| AGAP008118-PA [Anopheles gambiae str. PEST] |
| comp6467_c0_seq1 | PREDICTED: similar to conserved hypothetical protein [Tribolium castaneum] gi\|270006130\|gb\|EFA02578.1\| hypothetical protein TcasGA2_TC008296 [Tribolium castaneum] |
| comp5216_c0_seq1 | PREDICTED: similar to LD13710p [Nasonia vitripennis] |
| comp2540_c1_seq1 | conserved hypothetical protein [Pediculus humanus corporis] gi\|212508386\|gb\|EEB12098.1\| conserved hypothetical protein [Pediculus humanus corporis] |
| comp6233_c0_seq1 | PREDICTED: hypothetical protein LOC100649677 [Bombus terrestris] |
| comp1723_c0_seq1 | PREDICTED: probable nuclear transport factor 2-like isoform 3 [Apis mellifera] |
| comp5158_c0_seq1 | PREDICTED: similar to LOC447949 protein [Nasonia vitripennis] |
| comp5423_c0_seq1 | PREDICTED: peptidyl-prolyl cis-trans isomerase FKBP6-like isoform 4 [Acyrthosiphon pisum] gi\|328704816\|ref\|XP_003242614.1\| PREDICTED: peptidyl-prolyl cis-trans isomerase FKBP6-like isoform 2 [Acyrthosiphon pisum] &gt;gi\|328704818\|ref\|XP_003242615.1\| PREDICTED: peptidyl-prolyl cis-trans isomerase FKBP6-like isoform 3 [Acyrthosiphon pisum] |
| comp4715_c0_seq1 | PREDICTED: e3 ubiquitin-protein ligase TTC3-like [Xenopus (Silurana) tropicalis] |

**Table S2 |** Annotation of cabbage male bias genes (Cam up-regulated) relative to that of other host male.

| **Assembly ID** | **NR database** |
| --- | --- |
| comp1283_c3_seq3 | PREDICTED: hypothetical protein LOC100164870 [Acyrthosiphon pisum] |
| comp1895_c0_seq1 |  |
| comp3387_c0_seq1 | hsp70 [Bemisia tabaci] |
| comp4550_c0_seq1 |  |
| comp2838_c0_seq1 | RING finger protein, putative [Pediculus humanus corporis] gi\|212511290\|gb\|EEB14304.1\| RING finger protein, putative [Pediculus humanus corporis] |
| comp523_c0_seq2 |  |
| comp822_c0_seq1 | hypothetical protein TcasGA2_TC004472 [Tribolium castaneum] |
| comp3736_c0_seq5 |  |
| comp1138_c0_seq1 | multiple ankyrin repeats single kh domain protein, putative [Pediculus humanus corporis] gi\|212515433\|gb\|EEB17580.1\| multiple ankyrin repeats single kh domain protein, putative [Pediculus humanus corporis] |
| comp3584_c0_seq1 |  |
| comp3489_c0_seq12 | PREDICTED: ryanodine receptor 44F-like isoform 1 [Acyrthosiphon pisum] gi\|328717380\|ref\|XP_003246191.1\| PREDICTED: ryanodine receptor 44F-like isoform 2 [Acyrthosiphon pisum] |
| comp3613_c0_seq10 | PREDICTED: serine/threonine-protein kinase tricorner-like isoform 2 [Acyrthosiphon pisum] |
| comp3088_c0_seq1 |  |
| comp3173_c0_seq1 |  |
| comp2310_c0_seq17 |  |
| comp291_c1_seq2 | Hydroxymethylglutaryl-CoA synthase 1 [Acromyrmex echinatior] |
| comp9312_c0_seq1 |  |
| comp5607_c0_seq1 | heat shock protein 70 [Bemisia tabaci] |
| comp1086_c1_seq8 |  |
| comp7754_c0_seq1 |  |
| comp5185_c0_seq1 |  |
| comp2745_c0_seq1 |  |
| comp4157_c0_seq1 | coatomer alpha subunit, putative [Pediculus humanus corporis] gi\|212512887\|gb\|EEB15564.1\| coatomer alpha subunit, putative [Pediculus humanus corporis] |
| comp274_c0_seq1 | Actin-1 [Ascaris suum] |
| comp708_c0_seq2 |  |
| comp12696_c0_seq1 |  |
| comp16306_c0_seq1 | PREDICTED: A disintegrin and metalloproteinase with thrombospondin motifs 16-like isoform 2 [Acyrthosiphon pisum] |
| comp6010_c0_seq5 |  |
| comp10526_c0_seq2 | PREDICTED: similar to AGAP007137-PB [Tribolium castaneum] |
| comp2515_c0_seq5 |  |
| comp1459_c0_seq3 |  |
| comp663_c1_seq2 |  |
| comp634_c2_seq20 |  |
| comp3830_c0_seq1 |  |
| comp5864_c0_seq2 |  |
| comp3033_c0_seq1 | hypothetical protein TcasGA2_TC004196 [Tribolium castaneum] |
| comp624_c0_seq3 |  |
| comp6222_c0_seq1 | Protein ST7-like protein [Harpegnathos saltator] |
| comp7380_c0_seq1 | PREDICTED: similar to CG4713 CG4713-PA [Tribolium castaneum] |
| comp657_c0_seq4 |  |
| comp6072_c0_seq3 | hypothetical protein SINV_07775 [Solenopsis invicta] |
| comp9653_c0_seq1 |  |
| comp884_c0_seq1 | heat shock protein 70 [Bemisia tabaci] |
| comp2356_c0_seq1 | heat shock protein 70 [Spodoptera exigua] |
| comp234_c0_seq1 | ribosomal protein S27 [Xenopsylla cheopis] |
| comp987_c0_seq2 | heat shock protein 70 [Bemisia tabaci] |
| comp12821_c0_seq1 |  |
| comp5933_c0_seq1 | gram positive anchor [Streptococcus mitis SK1080] |
| comp2319_c0_seq7 |  |
| comp12746_c0_seq1 |  |
| comp6948_c0_seq1 |  |
| comp6908_c0_seq1 | hypothetical protein TcasGA2_TC012938 [Tribolium castaneum] |
| comp3291_c0_seq3 |  |
| comp10301_c0_seq1 |  |
| comp3616_c0_seq4 |  |
| comp522_c0_seq1 | Cathepsin B precursor [Lepeophtheirus salmonis] gi\|290561811\|gb\|ADD38303.1\| Cathepsin B [Lepeophtheirus salmonis] |
| comp1926_c0_seq4 |  |
| comp13171_c0_seq1 |  |
| comp8544_c0_seq2 | Chromatin-remodeling complex ATPase chain Iswi [Acromyrmex echinatior] |
| comp7673_c0_seq1 |  |
| comp7702_c0_seq1 |  |
| comp3736_c0_seq2 |  |
| comp4395_c0_seq1 |  |
| comp1747_c0_seq6 |  |
| comp2689_c0_seq4 | UDP-glucose:glycoprotein glucosyltransferase [Camponotus floridanus] |
| comp837_c0_seq1 |  |
| comp641_c0_seq7 |  |
| comp267_c0_seq1 | 60S ribosomal protein L37, putative [Pediculus humanus corporis] gi\|212506270\|gb\|EEB10530.1\| 60S ribosomal protein L37, putative [Pediculus humanus corporis] |
| comp6193_c0_seq2 |  |
| comp4370_c0_seq5 |  |
| comp3040_c0_seq1 | PREDICTED: hypothetical protein LOC551222 [Apis mellifera] |
| comp10311_c0_seq1 | PREDICTED: hypothetical protein LOC100161026 [Acyrthosiphon pisum] |
| comp336_c0_seq1 |  |
| comp6072_c0_seq2 |  |
| comp4007_c0_seq4 |  |
| comp1472_c0_seq1 |  |
| comp1562_c0_seq8 |  |
| comp3326_c1_seq2 | hypothetical protein TcasGA2_TC001390 [Tribolium castaneum] |
| comp6794_c0_seq1 |  |
| comp718_c0_seq5 |  |
| comp77_c6_seq87 |  |
| comp1524_c1_seq1 |  |
| comp6503_c0_seq3 |  |
| comp1807_c0_seq1 |  |
| comp2130_c0_seq1 | Putative hydroxyacid-oxoacid transhydrogenase, mitochondrial [Acromyrmex echinatior] |
| comp3367_c0_seq1 | PREDICTED: similar to ENSANGP00000031565 [Nasonia vitripennis] |
| comp2034_c0_seq1 |  |
| comp731_c0_seq3 |  |
| comp3168_c0_seq5 | unnamed protein product [Drosophila simulans] |
| comp3794_c0_seq3 |  |
| comp5546_c0_seq1 | PREDICTED: ATP-dependent helicase brm-like [Acyrthosiphon pisum] |
| comp1190_c0_seq1 |  |
| comp2022_c0_seq1 | small heat shock protein [Trialeurodes vaporariorum] |
| comp5729_c0_seq1 |  |
| comp6125_c0_seq1 |  |
| comp7710_c0_seq3 | PREDICTED: copia protein-like [Acyrthosiphon pisum] |
| comp5726_c0_seq2 |  |
| comp1375_c0_seq1 | heat shock protein 20 [Bemisia tabaci] gi\|295393288\|gb\|ADG03464.1\| heat shock protein 20 [Bemisia tabaci] &gt;gi\|295393294\|gb\|ADG03467.1\| heat shock protein 20 [Bemisia tabaci] &gt;gi\|308097851\|gb\|ADO14472.1\| hsp20 [Bemisia tabaci] |
| comp5947_c0_seq1 |  |
| comp3773_c0_seq1 |  |
| comp6473_c0_seq1 | similar to CG2708-PA [Papilio xuthus] |
| comp6036_c0_seq1 | PREDICTED: similar to voltage-gated calcium channel alpha2-delta subunit 1 [Tribolium castaneum] |
| comp5785_c0_seq1 | PREDICTED: MAU2 chromatid cohesion factor homolog [Acyrthosiphon pisum] |
| comp11442_c0_seq1 | PREDICTED: similar to structural maintenance of chromosomes 5 smc5 [Tribolium castaneum] gi\|270009507\|gb\|EFA05955.1\| hypothetical protein TcasGA2_TC008773 [Tribolium castaneum] |
| comp6756_c0_seq3 | Alpha-aminoadipic semialdehyde synthase, mitochondrial [Acromyrmex echinatior] |
| comp10816_c0_seq1 | PREDICTED: leucine-zipper-like transcription regulator 1 [Apis mellifera] |
| comp3341_c0_seq1 | PREDICTED: hypothetical protein LOC100165888 [Acyrthosiphon pisum] |
| comp2277_c0_seq1 | PREDICTED: acidic fibroblast growth factor intracellular-binding protein-like isoform 1 [Acyrthosiphon pisum] gi\|328715137\|ref\|XP_003245546.1\| PREDICTED: acidic fibroblast growth factor intracellular-binding protein-like isoform 2 [Acyrthosiphon pisum] |
| comp6238_c0_seq1 |  |
| comp9631_c0_seq1 | predicted protein [Populus trichocarpa] gi\|222871681\|gb\|EEF08812.1\| predicted protein [Populus trichocarpa] |
| comp7196_c0_seq1 | PREDICTED: hypothetical protein [Gallus gallus] |
| comp290_c1_seq1 | AGAP009783-PA [Anopheles gambiae str. PEST] gi\|157014010\|gb\|EAA14306.3\| AGAP009783-PA [Anopheles gambiae str. PEST] |
| comp1963_c0_seq2 | PREDICTED: similar to AGAP012587-PA [Tribolium castaneum] gi\|270004170\|gb\|EFA00618.1\| hypothetical protein TcasGA2_TC003494 [Tribolium castaneum] |
| comp291_c1_seq6 |  |
| comp591_c0_seq1 |  |
| comp11933_c0_seq2 | PREDICTED: fat-like cadherin-related tumor suppressor homolog isoform 2 [Acyrthosiphon pisum] |
| comp2175_c0_seq1 | hypothetical protein DAPPUDRAFT_310616 [Daphnia pulex] |
| comp20901_c0_seq1 | hypothetical protein SALIVA_1457 [Streptococcus salivarius JIM8777] |
| comp269_c0_seq1 |  |
| comp487_c0_seq2 | L-xylulose reductase [Anoplopoma fimbria] |
| comp8129_c0_seq1 |  |
| comp0_c0_seq1 |  |
| comp2319_c0_seq12 |  |
| comp7710_c0_seq5 | hypothetical protein TcasGA2_TC004282 [Tribolium castaneum] |
| comp5795_c0_seq1 |  |
| comp1101_c0_seq2 |  |
| comp5652_c0_seq1 |  |
| comp5481_c0_seq1 |  |
| comp440_c0_seq9 |  |
| comp322_c0_seq1 | PREDICTED: similar to putative cathepsin B-like proteinase [Tribolium castaneum] gi\|270012705\|gb\|EFA09153.1\| cathepsin B precursor [Tribolium castaneum] |
| comp1161_c0_seq1 |  |
| comp3476_c0_seq1 |  |
| comp715_c0_seq1 |  |
| comp5510_c0_seq1 | PREDICTED: nogo-B receptor-like [Acyrthosiphon pisum] gi\|239790664\|dbj\|BAH71880.1\| ACYPI27127 [Acyrthosiphon pisum] |
| comp8237_c1_seq1 |  |
| comp296_c0_seq4 |  |
| comp1390_c0_seq1 | KTI12 protein homolog [Acyrthosiphon pisum] gi\|239791240\|dbj\|BAH72113.1\| hypothetical protein [Acyrthosiphon pisum] |
| comp2256_c0_seq1 | PREDICTED: similar to glycinamide ribonucleotide synthetase-aminoimidazole ribonucleotide synthetase-glycinamide ribonucleotide transformylase [Tribolium castaneum] gi\|270011705\|gb\|EFA08153.1\| hypothetical protein TcasGA2_TC005772 [Tribolium castaneum] |
| comp7335_c0_seq1 | PREDICTED: PXMP2/4 family protein 4-like [Acyrthosiphon pisum] |
| comp1855_c0_seq3 |  |
| comp77_c6_seq67 |  |
| comp3771_c0_seq1 |  |
| comp5555_c0_seq3 |  |
| comp2319_c0_seq11 |  |
| comp7123_c0_seq2 |  |
| comp4567_c0_seq2 | Abhydrolase domain-containing protein 4 [Acromyrmex echinatior] |
| comp3641_c0_seq2 | Protein C1orf9-like protein [Acromyrmex echinatior] |
| comp5032_c0_seq1 | GfV-C17-ORF1 [Glypta fumiferanae ichnovirus] gi\|124270627\|dbj\|BAF45556.1\| GfV-C17-ORF1 [Glypta fumiferanae ichnovirus] |
| comp6660_c0_seq1 |  |
| comp2514_c0_seq1 | PREDICTED: probable maltase L-like [Acyrthosiphon pisum] |
| comp4540_c0_seq1 | PREDICTED: similar to CG3999 CG3999-PA [Tribolium castaneum] gi\|270004725\|gb\|EFA01173.1\| hypothetical protein TcasGA2_TC010496 [Tribolium castaneum] |
| comp2618_c0_seq5 | Hrp65 protein, putative [Pediculus humanus corporis] gi\|212515645\|gb\|EEB17750.1\| Hrp65 protein, putative [Pediculus humanus corporis] |
| comp1606_c0_seq3 | phosphatidylethanolamine-binding protein, putative [Ixodes scapularis] gi\|215510173\|gb\|EEC19626.1\| phosphatidylethanolamine-binding protein, putative [Ixodes scapularis] |
| comp6432_c0_seq2 | PREDICTED: hypothetical protein LOC100162709 isoform 3 [Acyrthosiphon pisum] |
| comp14706_c0_seq1 | PREDICTED: fibrillin-2-like [Acyrthosiphon pisum] |
| comp10846_c0_seq1 |  |
| comp6215_c0_seq7 |  |
| comp1087_c1_seq2 | PREDICTED: similar to ENSANGP00000027912 [Nasonia vitripennis] |
| comp2456_c0_seq1 | PREDICTED: signal recognition particle 68 kDa protein [Apis mellifera] |
| comp13026_c0_seq1 |  |
| comp13264_c0_seq1 | PREDICTED: peptide transporter family 1-like [Acyrthosiphon pisum] |
| comp758_c1_seq8 |  |
| comp1906_c0_seq2 | Nesprin-1 [Harpegnathos saltator] |
| comp5151_c0_seq1 | Autophagy-related protein 101 [Acromyrmex echinatior] |
| comp3803_c0_seq1 |  |
| comp2369_c0_seq4 |  |
| comp7372_c0_seq1 | PREDICTED: transforming growth factor beta regulator 1 isoform 1 [Nomascus leucogenys] |
| comp8632_c0_seq1 | PREDICTED: similar to methionine aminopeptidase [Tribolium castaneum] |
| comp11350_c1_seq1 |  |
| comp8026_c0_seq1 | PREDICTED: similar to enhancer of zeste homolog 2 [Tribolium castaneum] gi\|270003813\|gb\|EFA00261.1\| hypothetical protein TcasGA2_TC003094 [Tribolium castaneum] |
| comp5878_c0_seq1 | PREDICTED: similar to ovary C/EBPg transcription factor [Nasonia vitripennis] |
| comp11526_c0_seq1 | AGAP002737-PA [Anopheles gambiae str. PEST] |
| comp5825_c0_seq1 | PREDICTED: similar to zinc finger protein [Tribolium castaneum] gi\|270001517\|gb\|EEZ97964.1\| hypothetical protein TcasGA2_TC000356 [Tribolium castaneum] |
| comp2377_c0_seq1 |  |
| comp12037_c0_seq1 | PREDICTED: hypothetical protein LOC100569971 [Acyrthosiphon pisum] |
| comp2657_c0_seq1 |  |
| comp3_c0_seq34 | hypothetical protein VIBHAR_01012 [Vibrio harveyi ATCC BAA-1116] |
| comp2307_c0_seq1 | PREDICTED: G-protein coupled receptor Mth2-like [Acyrthosiphon pisum] |
| comp2628_c0_seq1 | PREDICTED: similar to conserved hypothetical protein [Nasonia vitripennis] |
| comp4682_c0_seq1 |  |
| comp11738_c0_seq1 | hypothetical protein Phum_PHUM294490 [Pediculus humanus corporis] gi\|212511365\|gb\|EEB14369.1\| hypothetical protein Phum_PHUM294490 [Pediculus humanus corporis] |
| comp14394_c0_seq2 |  |
| comp1192_c0_seq1 | PREDICTED: 6-phosphofructokinase-like isoform 2 [Acyrthosiphon pisum] |
| comp13694_c0_seq3 |  |
| comp12368_c0_seq1 | PREDICTED: similar to high-affinity octopamine transporter protein [Tribolium castaneum] |
| comp5730_c0_seq1 |  |
| comp3265_c0_seq1 | PREDICTED: similar to tumor endothelial marker 7 [Nasonia vitripennis] |
| comp8658_c0_seq2 |  |
| comp8322_c0_seq1 | EH domain-binding protein 1 [Acromyrmex echinatior] |
| comp1551_c1_seq2 | hypothetical protein TcasGA2_TC015690 [Tribolium castaneum] |
| comp14603_c0_seq1 |  |
| comp2041_c0_seq1 |  |
| comp3097_c0_seq1 |  |
| comp2376_c1_seq1 | hypothetical protein TcasGA2_TC013383 [Tribolium castaneum] |
| comp77_c6_seq63 | PREDICTED: similar to ENSANGP00000019430 [Nasonia vitripennis] |
| comp2463_c0_seq1 | PREDICTED: similar to conserved hypothetical protein [Nasonia vitripennis] |
| comp8152_c0_seq1 |  |
| comp347_c0_seq1 | ubiquinol-cytochrome c reductase complex [Xenopus (Silurana) tropicalis] gi\|89269531\|emb\|CAJ82412.1\| ubiquinol-cytochrome c reductase complex (7.2 kD) (ucrc) [Xenopus (Silurana) tropicalis] &gt;gi\|160774282\|gb\|AAI55049.1\| ubiquinol-cytochrome c reductase complex (7.2 kD) (ucrc) [Xenopus (Silurana) tropicalis] |
| comp5413_c0_seq1 | pre-mRNA-splicing factor ATP-dependent RNA helicase prp16, putative [Pediculus humanus corporis] gi\|212511335\|gb\|EEB14339.1\| pre-mRNA-splicing factor ATP-dependent RNA helicase prp16, putative [Pediculus humanus corporis] |
| comp8802_c0_seq1 | PREDICTED: similar to AGAP005207-PA [Tribolium castaneum] |
| comp5289_c2_seq1 |  |
| comp13008_c0_seq1 |  |
| comp3187_c0_seq1 |  |
| comp1087_c0_seq1 | AGAP001065-PA [Anopheles gambiae str. PEST] gi\|333469515\|gb\|EGK97320.1\| AGAP001065-PB [Anopheles gambiae str. PEST] &gt;gi\|333469516\|gb\|EGK97321.1\| AGAP001065-PC [Anopheles gambiae str. PEST] &gt;gi\|333469517\|gb\|EGK97322.1\| AGAP001065-PD [Anopheles gambiae str. PEST] &gt;gi\|333469518\|gb\|EGK97323.1\| AGAP001065-PE [Anopheles gambiae str. PEST] &gt;gi\|333469519\|gb\|EGK97324.1\| AGAP001065-PF [Anopheles gambiae str. PEST] &gt;gi\|333469520\|gb\|EGK97325.1\| AGAP001065-PG [Anopheles gambiae str. PEST] &gt;gi\|333469521\|gb\|EGK97326.1\| AGAP001065-PH [Anopheles gambiae str. PEST] |
| comp1083_c0_seq1 | hypothetical protein TcasGA2_TC014051 [Tribolium castaneum] |
| comp9281_c0_seq2 | hypothetical protein DAPPUDRAFT_49503 [Daphnia pulex] |
| comp923_c0_seq1 | PREDICTED: ribose-phosphate pyrophosphokinase 1-like isoform 1 [Bombus terrestris] |
| comp5975_c0_seq1 | PREDICTED: protein yippee-like 1-like [Acyrthosiphon pisum] |
| comp354_c0_seq3 | cathepsin B [Trichinella spiralis] gi\|316977649\|gb\|EFV60721.1\| cathepsin B [Trichinella spiralis] |
| comp4881_c0_seq1 |  |
| comp6971_c0_seq1 |  |
| comp684_c1_seq20 |  |
| comp7877_c0_seq1 | conserved hypothetical protein [Pediculus humanus corporis] gi\|212511325\|gb\|EEB14329.1\| conserved hypothetical protein [Pediculus humanus corporis] |
| comp2033_c0_seq7 | BTB/POZ domain-containing protein, putative [Pediculus humanus corporis] gi\|212518468\|gb\|EEB20221.1\| BTB/POZ domain-containing protein, putative [Pediculus humanus corporis] |
| comp3538_c0_seq1 |  |
| comp2135_c0_seq1 |  |
| comp929_c0_seq1 |  |
| comp7409_c0_seq1 | conserved hypothetical protein [Pediculus humanus corporis] gi\|212507988\|gb\|EEB11807.1\| conserved hypothetical protein [Pediculus humanus corporis] |
| comp7174_c0_seq1 | hypothetical protein TcasGA2_TC009369 [Tribolium castaneum] |
| comp4737_c0_seq2 |  |
| comp732_c0_seq2 | PREDICTED: flavin-containing monooxygenase FMO GS-OX3-like [Acyrthosiphon pisum] |
| comp1236_c0_seq1 | hypothetical protein SINV_05217 [Solenopsis invicta] |
| comp4596_c0_seq3 | PREDICTED: similar to AGAP006448-PB [Tribolium castaneum] gi\|270003022\|gb\|EEZ99469.1\| hypothetical protein TcasGA2_TC000040 [Tribolium castaneum] |
| comp9997_c0_seq1 |  |
| comp5477_c0_seq1 | cytochrome P450 [Bemisia tabaci] |
| comp10987_c0_seq1 |  |
| comp6100_c0_seq2 |  |
| comp4928_c1_seq2 | PREDICTED: myosin-IA-like [Bombus terrestris] |
| comp2043_c0_seq1 | hypothetical protein SINV_08588 [Solenopsis invicta] |
| comp3248_c0_seq1 |  |
| comp12409_c0_seq1 |  |
| comp82_c0_seq1 | NADH dehydrogenase subunit 2 [Bemisia tabaci] gi\|51944948\|gb\|AAU14204.1\| NADH dehydrogenase subunit II [Bemisia tabaci] |
| comp8507_c0_seq1 | rest corepressor corest, protein, putative [Pediculus humanus corporis] gi\|212514851\|gb\|EEB17095.1\| rest corepressor corest, protein, putative [Pediculus humanus corporis] |
| comp18_c0_seq1 | NADH dehydrogenase subunit 5 [Bemisia tabaci] gi\|51944941\|gb\|AAU14197.1\| NADH dehydrogenase subunit 5 [Bemisia tabaci] |
| comp1357_c0_seq1 |  |
| comp8165_c0_seq1 | parafibromin, putative [Pediculus humanus corporis] gi\|212512456\|gb\|EEB15219.1\| parafibromin, putative [Pediculus humanus corporis] |
| comp354_c0_seq1 | hypothetical protein BRAFLDRAFT_209578 [Branchiostoma floridae] gi\|229291947\|gb\|EEN62613.1\| hypothetical protein BRAFLDRAFT_209578 [Branchiostoma floridae] |
| comp5392_c0_seq1 |  |
| comp3345_c0_seq1 | PREDICTED: nuclear pore complex protein Nup98-Nup96-like [Bombus terrestris] |
| comp9669_c0_seq2 | RNA-directed RNA polymerase [Circulifer tenellus virus 1] gi\|300432047\|gb\|ADK12924.1\| RNA-directed RNA polymerase [Circulifer tenellus virus 1] |
| comp14061_c0_seq1 |  |
| comp2190_c0_seq1 | PREDICTED: similar to lysosomal alpha-mannosidase (mannosidase alpha class 2b member 1) [Tribolium castaneum] gi\|270003256\|gb\|EEZ99703.1\| hypothetical protein TcasGA2_TC002464 [Tribolium castaneum] |
| comp1315_c0_seq1 | PREDICTED: similar to phosphoribosylaminoimidazole carboxylase [Nasonia vitripennis] |
| comp77_c6_seq20 |  |
| comp778_c0_seq5 |  |
| comp1575_c0_seq1 | phosphoserine aminotransferase, putative [Pediculus humanus corporis] gi\|212516605\|gb\|EEB18599.1\| phosphoserine aminotransferase, putative [Pediculus humanus corporis] |
| comp5753_c0_seq2 |  |
| comp740_c0_seq1 | formate-tetrahydrofolate ligase, putative [Pediculus humanus corporis] gi\|212505864\|gb\|EEB10210.1\| formate-tetrahydrofolate ligase, putative [Pediculus humanus corporis] |
| comp4674_c0_seq1 | PREDICTED: similar to surfeit 4-like protein [Tribolium castaneum] gi\|270001352\|gb\|EEZ97799.1\| hypothetical protein TcasGA2_TC000161 [Tribolium castaneum] |
| comp6328_c0_seq5 | Serine/threonine-protein kinase D3 [Camponotus floridanus] |
| comp9779_c0_seq1 | PREDICTED: similar to conserved hypothetical protein [Nasonia vitripennis] |
| comp1342_c1_seq1 | PREDICTED: d-3-phosphoglycerate dehydrogenase-like [Apis mellifera] |
| comp8763_c0_seq1 | PREDICTED: similar to elongase, putative [Nasonia vitripennis] |
| comp1526_c0_seq3 |  |
| comp6387_c0_seq1 |  |
| comp4002_c0_seq4 |  |
| comp1483_c0_seq1 | Methylmalonic aciduria and homocystinuria type D-like protein, mitochondrial [Acromyrmex echinatior] |
| comp2085_c0_seq1 | PREDICTED: probable maltase H [Acyrthosiphon pisum] |
| comp6896_c0_seq1 | AGAP003371-PA [Anopheles gambiae str. PEST] |
| comp2932_c0_seq1 |  |
| comp3669_c1_seq1 | unnamed protein product [Drosophila melanogaster] |
| comp3899_c0_seq3 | PREDICTED: hypothetical protein [Nasonia vitripennis] |
| comp3301_c0_seq1 | hypothetical protein LOC100158934 [Acyrthosiphon pisum] gi\|239790868\|dbj\|BAH71968.1\| ACYPI000351 [Acyrthosiphon pisum] |
| comp1455_c0_seq1 |  |
| comp4442_c0_seq1 | GTP-binding protein Rheb-like [Acyrthosiphon pisum] gi\|239791527\|dbj\|BAH72216.1\| ACYPI008618 [Acyrthosiphon pisum] |
| comp6128_c0_seq1 | conserved hypothetical protein [Pediculus humanus corporis] gi\|212512083\|gb\|EEB14915.1\| conserved hypothetical protein [Pediculus humanus corporis] |
| comp4064_c0_seq6 | hypothetical protein TcasGA2_TC008225 [Tribolium castaneum] |
| comp5772_c0_seq1 |  |
| comp3681_c0_seq1 | PREDICTED: hypothetical protein [Nasonia vitripennis] |
| comp3416_c0_seq1 |  |
| comp4503_c0_seq2 | PREDICTED: short spindle protein 4-like isoform 2 [Bombus terrestris] |
| comp6562_c0_seq2 |  |
| comp1886_c0_seq1 | Calcium-independent phospholipase A2-gamma [Acromyrmex echinatior] |
| comp732_c0_seq1 | PREDICTED: flavin-containing monooxygenase FMO GS-OX3-like [Acyrthosiphon pisum] |
| comp634_c2_seq13 |  |
| comp1126_c0_seq1 |  |
| comp2002_c0_seq1 | clathrin coat associated protein ap-50 [Aedes aegypti] gi\|108881420\|gb\|EAT45645.1\| clathrin coat associated protein ap-50 [Aedes aegypti] |
| comp4809_c0_seq1 | PREDICTED: hypothetical protein LOC100160556 [Acyrthosiphon pisum] |
| comp5536_c0_seq1 | hypothetical protein TcasGA2_TC008263 [Tribolium castaneum] |
| comp1111_c2_seq3 | putative RNA-directed DNA polymerase (Reverse transcriptase) [Malus x domestica] |
| comp5049_c0_seq3 | PREDICTED: similar to AGAP005257-PA [Tribolium castaneum] |
| comp333_c0_seq1 |  |
| comp5020_c0_seq1 | Protein BTG3 [Camponotus floridanus] |
| comp7124_c0_seq2 | PREDICTED: integral membrane protein DGCR2/IDD-like [Apis mellifera] |
| comp476_c0_seq2 | SCP-related protein [Bombyx mori] gi\|110559661\|gb\|ABG76067.1\| SCP-related protein [Bombyx mori] |
| comp3778_c0_seq1 |  |
| comp10583_c0_seq1 |  |
| comp5731_c0_seq1 |  |
| comp1860_c0_seq1 | DnaJ-like protein subfamily C member 7 [Harpegnathos saltator] |
| comp2698_c0_seq8 | PREDICTED: la-related protein 1 [Apis mellifera] |
| comp4233_c0_seq1 |  |
| comp1331_c5_seq8 |  |
| comp3325_c0_seq1 | Protein FAM44A [Harpegnathos saltator] |
| comp1522_c0_seq1 |  |
| comp2096_c0_seq1 |  |
| comp2718_c0_seq1 | PREDICTED: short-chain specific acyl-CoA dehydrogenase, mitochondrial-like isoform 3 [Acyrthosiphon pisum] gi\|328705315\|ref\|XP_003242761.1\| PREDICTED: short-chain specific acyl-CoA dehydrogenase, mitochondrial-like isoform 2 [Acyrthosiphon pisum] |
| comp6461_c0_seq1 | Chondroitin sulfate synthase, putative [Pediculus humanus corporis] gi\|212507482\|gb\|EEB11419.1\| Chondroitin sulfate synthase, putative [Pediculus humanus corporis] |
| comp4971_c0_seq1 | Neuronal calcium sensor, putative [Pediculus humanus corporis] gi\|212516957\|gb\|EEB18911.1\| Neuronal calcium sensor, putative [Pediculus humanus corporis] |
| comp2925_c0_seq2 |  |
| comp3793_c0_seq1 | GK12980 [Drosophila willistoni] gi\|194169825\|gb\|EDW84726.1\| GK12980 [Drosophila willistoni] |
| comp2573_c0_seq1 |  |
| comp77_c6_seq37 |  |
| comp6194_c0_seq1 | PREDICTED: similar to tyrosine aminotransferase [Nasonia vitripennis] |

**Table S3 |** List of genes with Ka/Ks larger than one in Caf/Cof

| **Sequence ID** | **S-Sub** | **N-Sub** | **Ka** | **Ks** | **Ka/Ks** | **Nr or Swissprot annotation** |
| --- | --- | --- | --- | --- | --- | --- |
| Pool_comp8493_c0_seq1 | 1.9 | 21.1 | 0.0342201 | 0.0087352 | 3.91751 | Para sodium channel |
| Pool_comp9177_c0_seq4 | 1.0 | 11.0 | 0.0207891 | 0.0068171 | 3.04958 | Protein kinase C, brain isozyme-like |
| Pool_comp2751_c0_seq1 | 1.0 | 8.0 | 0.0024899 | 0.0009011 | 2.76326 | Conserved hypothetical protein |
| Pool_comp1843_c0_seq2 | 4.0 | 26.0 | 0.0724198 | 0.0391133 | 1.85154 | Centrin |
| Pool_comp2742_c0_seq3 | 1.0 | 4.0 | 0.0179213 | 0.0109988 | 1.62939 | Hypothetical protein |
| Pool_comp6714_c0_seq1 | 2.3 | 13.7 | 0.0090338 | 0.0055788 | 1.61931 | Hypothetical protein SINV_07643 |
| Pool_comp13790_c0_seq1 | 1.0 | 5.0 | 0.0153489 | 0.0107329 | 1.43008 | Unnamed protein product |
| Pool_comp16389_c0_seq1 | 1.3 | 4.7 | 0.0333563 | 0.0245458 | 1.35894 | Hypothetical protein SNOG_02847 |
| Pool_comp1192_c0_seq5 | 5.8 | 24.2 | 0.0171107 | 0.0130424 | 1.31193 | 6-phosphofructokinase, putative |
| Pool_comp1498_c1_seq1 | 1.0 | 4.0 | 0.0071385 | 0.0055527 | 1.28559 | Vacuolar ATP synthase subunit H, putative |
| Pool_comp2436_c0_seq1 | 4.6 | 21.4 | 0.0177608 | 0.0146069 | 1.21592 | Midasin-like |
| Pool_comp14021_c0_seq1 | 1.0 | 4.0 | 0.0211641 | 0.0176612 | 1.19834 | Hypothetical protein BRAFLDRAFT_66468 |
| Pool_comp8461_c0_seq1 | 11.1 | 43.9 | 0.0158895 | 0.0147805 | 1.07503 | Hypothetical protein TcasGA2_TC010195 |
| Pool_comp5115_c0_seq4 | 10.2 | 42.8 | 0.175213 | 0.163514 | 1.07155 | Hypothetical protein TcasGA2_TC003063 |
| Pool_comp9408_c0_seq1 | 5.0 | 22.0 | 0.10889 | 0.101774 | 1.06992 | Conserved hypothetical protein |
| Pool_comp1487_c0_seq1 | 5.0 | 11.0 | 0.0007092 | 0.0006688 | 1.06034 | Viral A-type inclusion protein |
| Pool_comp5903_c0_seq7 | 1.0 | 4.0 | 0.0131696 | 0.0125491 | 1.04944 | Nucleic-acid-binding protein from transposon X-element |
| Pool_comp32705_c0_seq1 | 2.0 | 8.0 | 0.0528077 | 0.050593 | 1.04377 | Similar to ENSANGP00000029084 |
| Pool_comp16382_c0_seq1 | 1.9 | 8.1 | 0.0147605 | 0.0141557 | 1.04272 | Solute carrier family 25 member 42-like isoform 1 |
| Pool_comp8651_c0_seq5 | 15.2 | 47.8 | 0.0088734 | 0.0086443 | 1.0265 | Filamin-C-like |
| Pool_comp15988_c0_seq1 | 3.0 | 9.0 | 0.0895257 | 0.0876054 | 1.02192 | Protein tamozhennic |
| Pool_comp4157_c0_seq1 | 12.7 | 41.3 | 0.0153276 | 0.0150286 | 1.01989 | Coatomer alpha subunit, putative |
| Pool_comp14175_c0_seq1 | 18.2 | 65.8 | 0.128619 | 0.126508 | 1.01668 | Cytochrome P450 |
| Pool_comp118_c0_seq1 | 2.4 | 6.6 | 0.0030133 | 0.0029757 | 1.01263 | Calcium-transporting ATPase sarcoplasmic |

**Table S4 |** List of genes with Ka/Ks larger than one in Caf/Cuf

| **Sequence ID** | **S-Sub** | **N-Sub** | **Ka** | **Ks** | **Ka/Ks** | **Nr or Swissprot annotation** |
| --- | --- | --- | --- | --- | --- | --- |
| Pool_comp11574_c0_seq1 | 1.0 | 15.0 | 0.0520759 | 0.0115315 | 4.51598 | Hypothetical protein LOC100571804 |
| Pool_comp9177_c0_seq4 | 1.0 | 11.0 | 0.0185316 | 0.0062408 | 2.96945 | Protein kinase C, brain isozyme-like |
| Pool_comp6239_c0_seq1 | 1.0 | 9.0 | 0.0168461 | 0.0059919 | 2.81147 | Similar to mrj CG8448-PA |
| Pool_comp20209_c0_seq1 | 1.0 | 6.0 | 0.0189925 | 0.007393 | 2.56899 | Predicted protein |
| Pool_comp8486_c0_seq1 | 2.2 | 14.8 | 0.0019217 | 0.0007519 | 2.55574 | Ankyrin repeat protein |
| Pool_comp9233_c0_seq1 | 1.0 | 5.0 | 0.0186002 | 0.0095906 | 1.93941 | Pecanex-like protein 1 |
| Pool_comp7718_c0_seq2 | 1.1 | 7.0 | 0.0526774 | 0.0322375 | 1.63404 | Armadillo segment polarity protein |
| Pool_comp362_c0_seq1 | 1.0 | 5.0 | 0.0014449 | 0.0010225 | 1.41311 | Conserved hypothetical protein |
| Pool_comp5517_c0_seq1 | 1.0 | 5.0 | 0.0045349 | 0.0032643 | 1.38923 | Hypothetical protein LOC100164472 |
| Pool_comp23646_c0_seq6 | 2.0 | 5.0 | 0.0110981 | 0.0085299 | 1.30109 | Hypothetical protein LOC411638 |
| Pool_comp1498_c1_seq1 | 1.0 | 4.0 | 0.0071385 | 0.0055527 | 1.28559 | Vacuolar ATP synthase subunit H |
| Pool_comp7143_c0_seq1 | 2.0 | 9.0 | 0.0064204 | 0.0052379 | 1.22575 | Similar to conserved hypothetical protein |
| Pool_comp31888_c0_seq1 | 3.0 | 9.0 | 0.032283 | 0.0293925 | 1.09834 | 2-deoxyglucose-6-phosphate phosphatase |
| Pool_comp7690_c0_seq1 | 1.0 | 3.0 | 0.005903 | 0.0054992 | 1.07343 | Similar to Rhythmically expressed gene 2 CG3200-PA |
| Pool_comp6641_c0_seq1 | 1.0 | 3.0 | 0.0024071 | 0.0022439 | 1.07272 | E3 ubiquitin-protein ligase LRSAM1 |
| Pool_comp18791_c0_seq1 | 2.0 | 6.0 | 0.0187362 | 0.0182915 | 1.02431 | Haloacid dehalogenase-like hydrolase domain containing 1A |
| Pool_comp3924_c0_seq2 | 1.0 | 3.0 | 0.0015218 | 0.0015158 | 1.00398 | Puromycin-sensitive aminopeptidase-like |

**Table S5 |** List of genes with Ka/Ks larger than one in Caf/Tof

| **Sequence ID** | **S-Sub** | **N-Sub** | **Ka** | **Ks** | **Ka/Ks** | **Nr or Swissprot annotation** |
| --- | --- | --- | --- | --- | --- | --- |
| Pool_comp11574_c0_seq1 | 1.0 | 15.0 | 0.0520759 | 0.0115315 | 4.51598 | Hypothetical protein LOC100571804 |
| Pool_comp8486_c0_seq1 | 2.2 | 14.8 | 0.0019217 | 0.0007519 | 2.55574 | Ankyrin repeat protein |
| Pool_comp11305_c0_seq5 | 1.0 | 6.0 | 0.0301007 | 0.0139994 | 2.15014 | Conserved hypothetical protein |
| Pool_comp3059_c0_seq1 | 1.0 | 5.0 | 0.0031704 | 0.0015039 | 2.10806 | Transforming acidic coiled-coil-containing protein 1 |
| Pool_comp1509_c0_seq1 | 1.0 | 7.0 | 0.0094922 | 0.0048053 | 1.97534 | Uridine diphosphate glucosyltransferase |
| Pool_comp8290_c0_seq3 | 1.0 | 4.0 | 0.0074204 | 0.0041653 | 1.78147 | Isoleucyl-tRNA synthetase, mitochondrial |
| Pool_comp16622_c0_seq1 | 1.0 | 4.0 | 0.0046662 | 0.0027555 | 1.69343 | Uridine diphosphate glucosyltransferase |
| Pool_comp13736_c0_seq1 | 1.0 | 4.0 | 0.0035676 | 0.0021235 | 1.68006 | Hypothetical protein AaeL_AAEL014444 |
| Pool_comp17062_c0_seq3 | 1.0 | 5.0 | 0.0099201 | 0.0061106 | 1.62342 | Hypothetical protein TcasGA2_TC014519 |
| Pool_comp7713_c0_seq1 | 1.0 | 4.0 | 0.0038478 | 0.0023947 | 1.60681 | Origin recognition complex subunit 1 |
| Pool_comp5903_c0_seq7 | 1.0 | 5.0 | 0.0168706 | 0.0116043 | 1.45382 | Nucleic-acid-binding protein from transposon X-element |
| Pool_comp8806_c0_seq1 | 1.0 | 4.0 | 0.0123272 | 0.0086484 | 1.42538 | GI24413 |
| Pool_comp33343_c0_seq1 | 1.0 | 3.0 | 0.022927 | 0.0181208 | 1.26523 | GK21669 |
| Pool_comp13917_c0_seq1 | 1.0 | 3.0 | 0.0071641 | 0.0057039 | 1.256 | Integrator complex subunit 1 |
| Pool_comp15463_c0_seq1 | 2.2 | 9.8 | 0.0125836 | 0.0108437 | 1.16045 | FHA-HIT |
| Pool_comp16446_c0_seq1 | 10.2 | 40.8 | 0.0406985 | 0.0361549 | 1.12567 | Similar to Hermansky-Pudlak syndrome 3 homolog |
| Pool_comp2789_c0_seq1 | 3.7 | 15.3 | 0.0303819 | 0.0270488 | 1.12323 | Src substrate cortactin-like |
| Pool_comp4809_c0_seq1 | 1.4 | 8.6 | 0.0014349 | 0.0012933 | 1.10945 | Hypothetical protein LOC100160556 |
| Pool_comp31888_c0_seq1 | 3.0 | 9.0 | 0.0295258 | 0.0275164 | 1.07302 | 2-deoxyglucose-6-phosphate phosphatase |
| Pool_comp4344_c0_seq1 | 1.0 | 3.0 | 0.0005813 | 0.0005431 | 1.07036 | RING finger protein 17-like |
| Pool_comp9378_c0_seq2 | 1.4 | 3.6 | 0.0232489 | 0.0221328 | 1.05043 | Hypothetical protein TcasGA2_TC004583 |
| Pool_comp7638_c0_seq6 | 21.0 | 65.0 | 0.186648 | 0.184471 | 1.0118 | Spectrin beta chain-like isoform 2 |
| Pool_comp15890_c0_seq1 | 30.3 | 109.7 | 0.0628827 | 0.0627466 | 1.00217 | E3 ubiquitin-protein ligase HERC2-like |

**Table S6 |** List of genes with Ka/Ks larger than one in Cam/Com

| **Sequence ID** | **S-Sub** | **N-Sub** | **Ka** | **Ks** | **Ka/Ks** | **Nr or Swissprot annotation** |
| --- | --- | --- | --- | --- | --- | --- |
| Pool_comp7144_c0_seq2 | 3.3 | 38.7 | 0.195219 | 0.0895419 | 2.1802 | Laminin subunit alpha-like |
| Pool_comp8290_c0_seq3 | 1.0 | 5.0 | 0.0107502 | 0.0055101 | 1.951 | Isoleucyl-tRNA synthetase, mitochondrial |
| Pool_comp8194_c0_seq1 | 1.3 | 6.7 | 0.0449125 | 0.0299277 | 1.5007 | Proline-rich protein PRCC-like isoform 1 |
| Pool_comp6510_c0_seq1 | 1.0 | 4.0 | 0.021244 | 0.0144368 | 1.47152 | Cullin |
| Pool_comp362_c0_seq1 | 1.0 | 5.0 | 0.0014449 | 0.0010225 | 1.41311 | Conserved hypothetical protein |
| Pool_comp7643_c1_seq4 | 1.0 | 6.0 | 0.0122207 | 0.0090724 | 1.34701 | Copia protein-like |
| Pool_comp1275_c0_seq2 | 1.0 | 4.0 | 0.0113409 | 0.0084876 | 1.33617 | Hypothetical protein SINV_16053 |
| Pool_comp8493_c0_seq1 | 25.6 | 96.4 | 0.0635124 | 0.0543053 | 1.16954 | Para sodium channel |
| Pool_comp2789_c0_seq1 | 8.8 | 30.2 | 0.0546085 | 0.0539159 | 1.01285 | Src substrate cortactin-like |

**Table S7 |** List of genes with Ka/Ks larger than one in Cam/Cum

| **Sequence ID** | **S-Sub** | **N-Sub** | **Ka** | **Ks** | **Ka/Ks** | **Nr or Swissprot annotation** |
| --- | --- | --- | --- | --- | --- | --- |
| Pool_comp4691_c0_seq3 | 1.6 | 15.4 | 0.0489353 | 0.0152634 | 3.20606 | Serine protease nudel |
| Pool_comp19087_c0_seq3 | 1.6 | 12.4 | 0.0364698 | 0.0162526 | 2.24393 | Hypothetical protein LOC100161789 |
| Pool_comp8851_c0_seq1 | 1.0 | 3.0 | 0.0474143 | 0.0333157 | 1.42318 | AGAP011504-PA |
| Pool_comp7484_c0_seq2 | 1.0 | 4.0 | 0.0139266 | 0.0100666 | 1.38345 | Alanine aminotransferase 2-like |
| Pool_comp11933_c0_seq1 | 3.4 | 12.6 | 0.0039843 | 0.0030558 | 1.30387 | CG7749-PA |
| Pool_comp11747_c0_seq2 | 1.5 | 4.5 | 0.0076613 | 0.0059333 | 1.29125 | Tryptophan 5-hydroxylase 1-like |
| Pool_comp17398_c0_seq7 | 1.0 | 6.0 | 0.0280551 | 0.0237185 | 1.18284 | hypothetical protein TcasGA2_TC002266 |
| Pool_comp3345_c0_seq2 | 14.1 | 44.9 | 0.0963669 | 0.08476 | 1.13694 | Nuclear pore complex protein Nup98-Nup96-like |
| Pool_comp11368_c0_seq1 | 8.4 | 35.6 | 0.100927 | 0.0906972 | 1.11279 | Monocarboxylate transporter 10-like |
| Pool_comp5660_c0_seq1 | 1.0 | 3.0 | 0.0123835 | 0.0113888 | 1.08734 | Phosphatidylethanolamine-binding protein |
| Pool_comp17970_c0_seq1 | 3.8 | 11.2 | 0.0527037 | 0.0491305 | 1.07273 | SCF apoptosis response protein |
| Pool_comp8688_c0_seq1 | 14.4 | 40.6 | 0.0191396 | 0.0188038 | 1.01786 | Hect E3 ubiquitin ligase, putative |
| Pool_comp1232_c0_seq1 | 3.9 | 16.1 | 0.0071425 | 0.0071396 | 1.00041 | Zinc finger protein 658-like |

**Table S8 |** List of genes with Ka/Ks larger than one in Cam/Tom

| **Sequence ID** | **S-Sub** | **N-Sub** | **Ka** | **Ks** | **Ka/Ks** | **Nr or Swissprot annotation** |
| --- | --- | --- | --- | --- | --- | --- |
| Pool_comp9351_c0_seq1 | 1.0 | 10.0 | 0.0311213 | 0.0092071 | 3.38015 | Transcription elongation factor B polypeptide 3-like |
| Pool_comp13960_c0_seq1 | 3.7 | 32.3 | 0.0856269 | 0.0301733 | 2.83784 | Sn1-specific diacylglycerol lipase alpha-like |
| Pool_comp15213_c0_seq1 | 2.0 | 17.0 | 0.0281998 | 0.010283 | 2.74236 | Mitochondrial ubiquitin ligase activator of nfkb 1-like |
| Pool_comp5550_c1_seq1 | 1.0 | 9.0 | 0.0210564 | 0.0094458 | 2.22917 | Torso-like protein-like isoform 2 |
| Pool_comp48890_c0_seq1 | 1.6 | 11.4 | 0.0428978 | 0.0193691 | 2.21475 | Transposase |
| Pool_comp4867_c0_seq3 | 1.2 | 7.8 | 0.024984 | 0.0114459 | 2.1828 | UPF0489 protein C5orf22 homolog |
| Pool_comp3759_c0_seq2 | 1.0 | 9.0 | 0.0090072 | 0.0046563 | 1.93441 | Hypothetical protein TcasGA2_TC009548 |
| Pool_comp11890_c0_seq2 | 4.6 | 27.4 | 0.0218561 | 0.0116749 | 1.87205 | Forked protein, putative |
| Pool_comp8290_c0_seq3 | 1.0 | 4.0 | 0.0087711 | 0.0047828 | 1.83391 | Isoleucyl-tRNA synthetase, mitochondrial |
| Pool_comp5778_c0_seq1 | 3.6 | 29.4 | 0.0049414 | 0.0027619 | 1.78915 | No annotation |
| Pool_comp12801_c0_seq1 | 1.0 | 4.0 | 0.0047643 | 0.0027355 | 1.74164 | Aminopeptidase N precursor, putative |
| Pool_comp1232_c0_seq1 | 3.4 | 19.6 | 0.0103287 | 0.0061117 | 1.68999 | Zinc finger protein 658-like |
| Pool_comp8657_c0_seq3 | 3.1 | 11.9 | 0.012843 | 0.008598 | 1.49371 | Ankyrin |
| Pool_comp3691_c0_seq2 | 1.4 | 4.6 | 0.0063464 | 0.0043462 | 1.4602 | V-type proton ATPase 116 kDa subunit a isoform 1-like |
| Pool_comp17669_c0_seq1 | 1.1 | 3.9 | 0.0254337 | 0.0178878 | 1.42185 | Chondroitin sulfate synthase 2-like |
| Pool_comp362_c0_seq1 | 1.0 | 5.0 | 0.0014449 | 0.0010225 | 1.41311 | Conserved hypothetical protein |
| Pool_comp2231_c0_seq1 | 1.0 | 4.0 | 0.0016189 | 0.0011529 | 1.40413 | Hypothetical protein LOC100160261 |
| Pool_comp13262_c0_seq1 | 1.0 | 7.0 | 0.0223839 | 0.0159918 | 1.39972 | UPF0195 protein CG30152 |
| Pool_comp4858_c0_seq2 | 1.3 | 4.7 | 0.0159113 | 0.0122322 | 1.30077 | Ubiquitin carboxyl-terminal hydrolase DUB-1, putative |
| Pool_comp3759_c0_seq2 | 1.4 | 5.6 | 0.0011354 | 0.0008928 | 1.27171 | Hypothetical protein TcasGA2_TC009548 |
| Pool_comp11701_c0_seq3 | 2.0 | 7.0 | 0.0111988 | 0.0091818 | 1.21968 | Protoporphyrinogen IX oxidase, putative |
| Pool_comp16382_c0_seq1 | 12.7 | 56.3 | 0.0724901 | 0.0602147 | 1.20386 | Solute carrier family 25 member 42-like isoform 1 |
| Pool_comp6808_c0_seq1 | 1.0 | 3.0 | 0.0110147 | 0.0092009 | 1.19713 | Myosin IIIA |
| Pool_comp4748_c0_seq1 | 1.0 | 3.0 | 0.0026902 | 0.0022482 | 1.19658 | L-asparaginase i |
| Pool_comp8581_c0_seq2 | 1.9 | 6.1 | 0.0030996 | 0.0026288 | 1.17909 | Cathepsin F-like cysteine protease |
| Pool_comp36751_c0_seq1 | 2.5 | 6.5 | 0.0491641 | 0.0422002 | 1.16502 | Mitogen-activated protein kinase ERK-A, putative |
| Pool_comp11368_c0_seq1 | 5.7 | 23.3 | 0.0743846 | 0.0643064 | 1.15672 | Monocarboxylate transporter 10-like |
| Pool_comp29403_c0_seq1 | 21.1 | 57.9 | 0.437923 | 0.380468 | 1.15101 | Down syndrome cell adhesion molecule |
| Pool_comp12326_c0_seq2 | 13.9 | 84.1 | 0.0194204 | 0.0170169 | 1.14124 | Predicted protein |
| Pool_comp3491_c0_seq1 | 1.0 | 3.0 | 0.0025361 | 0.0022268 | 1.13892 | Cytochrome P450 |
| Pool_comp10971_c0_seq2 | 4.0 | 12.0 | 0.0035522 | 0.0031224 | 1.13764 | Neurogenic locus Notch protein |
| Pool_comp16726_c0_seq1 | 3.3 | 10.7 | 0.0119683 | 0.0106867 | 1.11992 | Sel1l protein |
| Pool_comp21267_c0_seq1 | 1.0 | 4.0 | 0.0230148 | 0.021107 | 1.09039 | GG15353 |
| Pool_comp12685_c0_seq1 | 1.0 | 3.0 | 0.0037926 | 0.0035341 | 1.07315 | Hypothetical protein LOC100644735 |
| Pool_comp13790_c0_seq1 | 1.0 | 3.0 | 0.0053185 | 0.0049754 | 1.06896 | Retrovirus-related Pol polyprotein from transposon TNT 1-94 |
| Pool_comp19944_c0_seq2 | 1.0 | 3.0 | 0.0058837 | 0.0056437 | 1.04253 | Lysosomal acid phosphatase precursor, putative |

**Table S9 |** Annotation and expression level in RNA sequencing of 17 up-regulated genes no matter in cabbage female or in male comparative to that of other host female or male, respectively.

|  | **RNA sequencing** | | | | | | |  |  |
| --- | --- | --- | --- | --- | --- | --- | --- | --- | --- |
|  | **log2(Fold_change) normalized** | | | | | | |  |  |
|  | **Gene ID** | **Caf/Cof** | **Caf/Cuf** | **Caf/Tof** | **Cam/Com** | **Cam/Cum** | **Cam/Tom** | **Average** | **Abbreviation_annotation** |
| 1 | comp822_c0_seq1 | 2.80 | 4.57 | 4.50 | 2.12 | 4.37 | 7.90 | 4.4 | Hypothetical protein_1 |
| 2 | comp9312_c0_seq1 | 3.71 | 2.45 | 1.90 | 3.43 | 3.10 | 6.68 | 3.5 |  |
| 3 | comp6908_c0_seq1 | 3.00 | 1.72 | 2.24 | 3.67 | 5.20 | 4.71 | 3.4 | Hypothetical protein_2 |
| 4 | comp522_c0_seq1 | 5.77 | 1.11 | 3.30 | 5.50 | 3.91 | 4.57 | 4.0 | Cathepsin B |
| 5 | comp2175_c0_seq1 | 2.54 | 2.33 | 1.48 | 3.07 | 1.78 | 2.68 | 2.3 | Hypothetical protein_3 |
| 6 | comp0_c0_seq1 | 2.48 | 1.40 | 1.24 | 1.49 | 4.92 | 2.58 | 2.4 |  |
| 7 | comp322_c0_seq1 | 5.76 | 1.63 | 3.44 | 7.38 | 1.85 | 2.48 | 3.8 | Cathepsin B like |
| 8 | comp5510_c0_seq1 | 2.58 | 6.26 | 2.03 | 2.97 | 6.85 | 2.47 | 3.9 | Nogo-B receptor-like |
| 9 | comp4567_c0_seq2 | 1.98 | 2.50 | 1.13 | 3.17 | 2.24 | 2.37 | 2.2 | Abhydrolase protein 4 |
| 10 | comp5413_c0_seq1 | 1.83 | 2.18 | 2.26 | 2.03 | 1.46 | 1.78 | 1.9 | ATP-dependent RNA helicase prp16 |
| 11 | comp2135_c0_seq1 | 2.32 | 1.00 | 1.71 | 2.41 | 1.81 | 1.63 | 1.8 |  |
| 12 | comp82_c0_seq1 | 3.82 | 3.24 | 4.89 | 1.84 | 7.20 | 1.51 | 3.7 | NADH dehydrogenase subunit 2 |
| 13 | comp18_c0_seq1 | 2.55 | 1.91 | 2.18 | 1.57 | 4.85 | 1.49 | 2.4 | NADH dehydrogenase subunit 5 |
| 14 | comp1357_c0_seq1 | 5.10 | 1.71 | 1.08 | 3.78 | 4.76 | 1.49 | 3.0 |  |
| 15 | comp5753_c0_seq2 | 2.16 | 1.05 | 1.97 | 2.88 | 3.84 | 1.40 | 2.2 |  |
| 16 | comp1455_c0_seq1 | 3.37 | 1.32 | 2.12 | 3.46 | 6.05 | 1.28 | 2.9 |  |
| 17 | comp4442_c0_seq1 | 2.72 | 1.17 | 2.22 | 3.15 | 4.28 | 1.28 | 2.5 | GTP-binding protein Rheb-like |

**Table S10 |** Primers used for the qRT-PCR analyses.

| **Number** | **Gene ID** | **Abbreviation_annotation** | **F** | **R** |
| --- | --- | --- | --- | --- |
| 1 | comp822_c0_seq1 | Hypothetical protein_1 | TTCTGGTTTGGCGTAGGTT | GCATCGCTCACAGCATCA |
| 2 | comp6908_c0_seq1 | Hypothetical protein_2 | TCACAACAGCAAATGCGAGAC | CACCCAAGCTCATCACCTACC |
| 3 | comp522_c0_seq1 | Cathepsin B | AACACGAAATCTCCCACCAT | ACCAGCCAATAGCCAACG |
| 4 | comp322_c0_seq1 | Cathepsin B like | GAGCACGAGGGCGTATCT | TCCGACTGTGGACTTGGTT |
| 5 | comp5510_c0_seq1 | Nogo-B receptor-like | TGTGGTGCGTTGGAAGC | CCGTTGTGAGGCTGTGC |
| 6 | comp4567_c0_seq2 | Abhydrolase protein 4 | GGCGTAAACAGGTCGAGTAG | CTGAACACGGAGTCAAAGAAA |
| 7 | comp5413_c0_seq1 | ATP-dependent RNA helicase prp16 | TCTGAAGAGCGTCCATACCG | GCGTCGTGGCTACCAACAT |
| 8 | comp82_c0_seq1 | NADH dehydrogenase subunit 2 | TAACCCTATCAACCTACCGAAA | TTGATTGATTTTAATCTGTGGC |
| 9 | comp18_c0_seq1 | NADH dehydrogenase subunit 5 | GGATATGCTCGGCAGGATTGT | TTATCGCTGTAGGGGTTATTGAGA |
| 10 | comp4442_c0_seq1 | GTP-binding protein Rheb-like | CCTTGTGGATACTGCGGGAC | GCACCAGCGGAACGTGAA |
|  | RPL29 |  | TCGGAAAATTACCGTGAG | GAACTTGTGATCTACTCCTCTCGTG |

**Table S11 | Validation and** **quantification of 10 up-regulated gene transcripts levels in original cabbage host according to the ∆CT method using *RPL29* as the reference gene (±S.E).** Genes were considered significantly over- or under-transcribed when the ∆CT values of RNA samples from cotton, cucumber and tomato plants were different from ∆CT values of RNA samples from cabbage in the two-sample *t* test.

| Plant | Hypothetical protein_1 | Hypothetical protein_2 | Cathepsin B | Cathepsin B like | Nogo-B receptor-like | Abhydrolase protein 4 | ATP-dependent RNA helicase prp16 | NADH dehydrogenase subunit 2 | NADH dehydrogenase subunit 5 | GTP-binding protein  Rheb-like |
| --- | --- | --- | --- | --- | --- | --- | --- | --- | --- | --- |
| **Cabbage** | 4.87±0.41 | 6.26±0.33 | 2.34±0.25 | -0.31±0.18 | 8.05±0.10 | 4.94±0.18 | 8.41±0.30 | -5.31±0.34 | -4.22±0.33 | 5.95±0.29 |
| Cucumber | 5.14±0.62 | 4.64±0.10^**^ | 6.46±0.45^##^ | 5.49±0.67^##^ | 7.22±0.21^*^ | 7.12±0.23^##^ | 7.35±0.41 | -3.38±0.42^#^ | -2.56±0.45^#^ | 3.70±0.20^**^ |
| Tomato | 3.27±0.39^*^ | 4.52±0.67 | 5.98±0.40^##^ | 6.20±0.25^##^ | 7.37±0.54 | 9.21±0.67^##^ | 9.10±0.75 | -3.14±0.08^##^ | -1.98±0.05^##^ | 4.57±0.17^*^ |
| Cotton | 2.42±0.36^*^ | 6.30±0.27 | 5.36±0.20^##^ | 3.75±0.29^##^ | 7.59±0.54 | 5.34±0.42 | 6.82±0.33^*^ | -4.39±0.57 | -3.88±0.35 | 3.87±0.17^**^ |

“^*^”: significantly up-transcribed in cucumber, tomato or cotton host strains compared to that in original cabbage host (*P*<0.05).

“^**^”: significantly up-transcribed in cucumber, tomato or cotton host strains compared to that in original cabbage host (*P*<0.01).

“^#^”: significantly under-transcribed in cucumber, tomato or cotton host strains compared to that in original cabbage host (*P*<0.05).

“^##^”: significantly under-transcribed in cucumber, tomato or cotton host strains compared to that in original cabbage host (*P*<0.01).
